# Multi-stimuli-responsive aggregation-induced emission of boryl substituted phenothiazine†

**DOI:** 10.1039/d5ra01331c

**Published:** 2025-05-12

**Authors:** Guoqiang Li, Yan Wang, Yaohui Li, Zengheng Wen, Zhuang Luo, Weijun Song, Weidong Zhang

**Affiliations:** a School of Chemical Engineering, Qinghai University Xining 810016 China zhangwd@qhu.edu.cn songweijun1980@163.com

## Abstract

Stimuli-responsive materials, especially multi-stimuli-responsive ones, represent a kind of intelligent materials with significant potential in high-tech innovations, owing to their ability to undergo physical property changes in response to external stimuli. This investigation produced three new functionalized donor–acceptor (D–A) fluorophores, specifically aminoboranes incorporating phenothiazyl groups (4a, 4b, and 4c), featuring analogous structural components. These aminoborane derivatives demonstrated excellent resistance to air/moisture degradation, along with reduced HOMO energy states compared to a CN-containing analog, 4d. Analysis indicated that these aminoborane compounds displayed fascinating photophysical characteristics, encompassing aggregation-induced emission (AIE). Notably, the diarylboryl-phenothiazines show case reversible and distinct multi-stimuli-responsive luminescence upon exposure to fluoride ions, voltage, and mechanical force. This investigation enhances understanding of molecular interaction mechanisms and structural modifications essential for developing advanced stimuli-responsive luminescent compounds.

## Abstract 2

A series of multi-stimuli-responsive luminophores, *i.e.*, aminoboranes containing a phenothiazyl substituent 4a, 4b, and 4c were synthesized and characterized.

## Graphical abstract

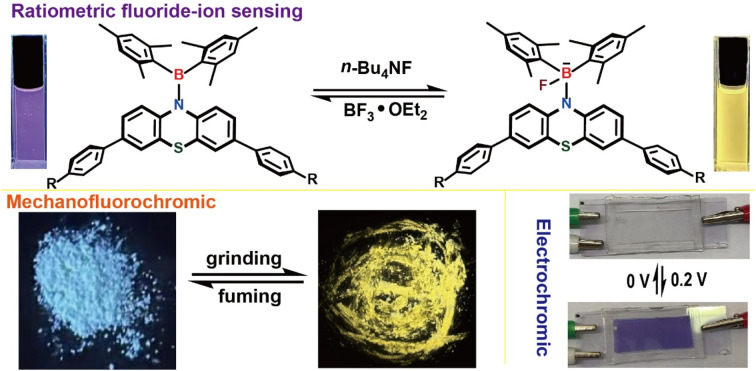

## Introduction

Functional materials that are stimuli-responsive have attracted considerable attention due to their ability to change color, fluorescence, and polarity in response to various external stimuli, including ions, solvent, gas, pH, heat, irradiation, and mechanical stress, endowing them with significant potential for application in chemical sensors, data storage devices, biomedicine, organic photovoltaics, light-emitting diodes, electrochromic devices, security papers and additional domain.^1–4^ However, most of the developed stimuli-responsive materials are designed for single-stimulus responses, exhibiting color or emission changes only in reaction to one specific stimulus, such as thermochromism, photochromism, mechanochromism, and electrochromism.^5,6^ Such limitations fail to satisfy the demand for more precise technologies and achieve diverse practical applications. Therefore, the development of multi-stimuli responsive (MSR) materials is not only of academic interest but also holds substantial practical value.

Recently, aggregation-induced emission (AIE), which demonstrates strong emission in the solid-state or aggregated state, has garnered significant attention as an efficient strategy for constructing solid-state emissive materials.^7–10^ Depending on the propeller-shaped structure and the restriction of intramolecular motions (RIM) influenced by their surrounding environment, most AIE molecules are packed loosely in the aggregation state or solid state. This packing allows for easy structural adjustments under various stimuli, leading to emission color changes upon stimulation.^11–13^ However, developing new MSR luminescent materials based on AIE remains largely dependent on random screening due to the mechanisms' diversity. Consequently, establishing a versatile strategy for AIE-type MSR luminescent materials, particularly those with high sensitivity to various external stimuli, remains a significant challenge. Notably, the MSR behavior of AIE molecules can be modulated through donor (D)–acceptor (A) substitutions in small organic molecules. The resulting twisted structures and stimuli-induced exciton coupling enhance fluorescence contrast and enable reversible changes under external stimuli, benefiting both locally excited (LE) and charge transfer (CT) states.^14,15^

The phenothiazine (PTZ) moiety represents a distinctive electron-abundant tricyclic heteroarene *versus* the tetraphenylethylene (TPE), distinguished by the incorporation of potent electron donors (D), specifically nitrogen (N) and sulphur (S) atoms.^16,17^ Furthermore, the non-planar butterfly-like conformation of the PTZ structure induces significant twisting, contributing to enhanced luminescence, high photoconductivity, and reversible oxidation, establishing it as a promising candidate for emission in solid or aggregated forms.^18–20^ The effectiveness of PTZ-incorporated optoelectronic components stems from the relatively steady, heterocyclic arrangement of their persistent radical cation.^21,22^ The incorporation of acceptor (A) substituents *via* π-extended conjugation at positions 3 or 7 of PTZ as the core of a D–A type AIE structure enhances intramolecular charge transfer (ICT), promoting exceptional stimuli-responsive behaviour. The trigonal-planar geometry and empty p_z_-orbital of three-coordinate organoboron compounds, which are electron-deficient, position them as strong acceptors (A) in conjugated organic π-systems.^23^ Nevertheless, the sensitivity to air and moisture exhibited by tricoordinate organoboranes, particularly boroles,^24,25^ presents a challenge for meeting the demands of high-performance optoelectronic devices.^26^ To address this issue, strategies to stabilize organoboranes have been reported, encompassing (1) attachment of sterically demanding aryl groups to coordinate the boron (B) centre and (2) substitution of the boron unit through BN-containing moiety in aromatic structures.^27–29^ BN-containing derivatives exhibit both improved stability and distinctive optical and electronic characteristics, finding applications in organic light emitting diodes, organic field effect transistor, display systems, sensing, and energy technology,^30–35^ while demonstrating large twisting angles, multiple intermolecular interactions, leading to highly enhanced emissions in aggregated states.^36,37^

To exploit and further development of the excellent optical properties of aggregated states, coupled with non-planar configuration and demanding D–A structural features, the electron-deficient boron unit (A) and electron-rich phenothiazine moieties (D) were selected as the core for constructing AIE structures. These structures were thoroughly investigated under various external stimuli, including fluoride ions, voltage, and mechanical force.

## Experimental section

### Materials and instrumentation

The anhydrous THF was distilled under sodium and benzophenone in argon atmosphere prior to application. The anhydrous DCM received drying treatment with calcium hydride followed by distillation. All experimental procedures were executed in an argon condition utilizing conventional Schlenk and glovebox methodologies, except as otherwise noted. Pd(Ph_3_P)_4_ (99%), *p*-tolylboronic acid (99%), (4-(trifluoromethyl)phenyl)boronic acid (99%), (4-methoxyphenyl)boronic acid (99%), potassium carbonate (99%), bromoethane (99%) and *n*-BuLi were procured from Anhui Zesheng Technology Co., Ltd. Compound 2 was synthesized utilizing reported protocols^38^ and 3a, 3b, and 3c was procured through established methods.^39^

Nuclear magnetic resonance spectra (NMR) were recorded using a Bruker-Avance III (^1^H: 600 MHz, ^13^C: 150 MHz) instrument with specified solvents; chemical shifts are expressed in units (ppm) by designating TMS signal in the ^1^H spectrum as 7.26 ppm, chloroform-*d* signal in the ^13^C spectrum as 77.0 ppm and dimethyl sulfoxide-d_6_ as 2.50 ppm in the ^1^H spectrum and 39.52 ppm in ^13^C spectrum. UV-vis data were obtained utilizing a DH-2000-BAL system. Emission data were acquired on an FLS920 and MicroTEQ-R1 system of ocean optics. Electrochemistry was performed utilizing a Metrohm PGSTAT204 setup.

The theoretical calculation investigations presented in this investigation were executed through the Gaussian 09 program. The ground state configurations of these molecules underwent optimization utilizing the B3LYP[3,4]-D3 hybrid unit.

### Experimental procedures

#### 10-(Dimesitylboraneyl)-3,7-di-*p*-tolyl-10*H*-phenothiazine (4a)

Compound 4a was synthesized per the established protocols.^40,41^ In an argon atmosphere, 3a (1.90 g, 5.0 mmol) was introduced into 30 mL anhydrous tetrahydrofuran under −78°, followed by dropwise addition of *n*-butyllithium solution (2.5 M in hexanes, 6.0 mmol) *via* syringe. The reaction solution was maintained at −78° for 30 min, succeeded by dropwise incorporation of Mes_2_BF (1.41 g, 5.27 mmol) in THF solution at −78°. The reaction mixture reached rt and stirred for 12 h. Following solvent evaporation under vacuum, the resulting mixture underwent extraction with DCM (60 mL × 3). Upon solvent evaporation under diminished pressure, the resulting crude material was subjected to purification through silica gel chromatography using PE/DCM (5 : 1 v/v) as eluent, yielding a white solid. Yield: 2.38 g (76%). ^1^H NMR (600 MHz, CDCl_3_): *δ* 7.49 (s, 2H, Ar*H*), 7.40 (d, 2H, *J* = 7.8 Hz, Ar*H*), 7.19 (t, 6H, *J* = 7.2 Hz, Ar*H*), 7.10 (d, 2H, *J* = 8.4 Hz, Ar*H*), 6.67 (s, 4H, Ar*H*), 2.40 (s, 12H, C*H*_3_), 2.36 (s, 6H, C*H*_3_), 2.19 (s, 6H, C*H*_3_); ^13^C NMR (151 MHz, CDCl_3_): *δ* 142.48, 140.63, 137.88, 137.20, 137.17, 136.68, 129.91, 129.46, 128.06, 126.59, 125.41, 125.28, 124.95, 23.93, 21.09, 20.98. HRMS (APCI) *m*/*z*: [M + H]^+^ calcd for C_44_H_43_BNS, 628.3209, found, 628.3190. Anal. calcd For C_44_H_42_BNS: C, 84.19; H, 6.74; N, 2.23. Found: C, 84.12; H, 6.68; N, 2.19. Fluorescence emission (in solid) (*λ*_ex_ = 360 nm): *λ*_emis_ = 434 nm (*Φ* = 0.16; *τ* = 5.92 ns).

#### 10-(Dimesitylboraneyl)-3,7-bis(4-methoxyphenyl)-10*H*-phenothia-zine (4b)

Compound 4b was synthesized using an analogous method to prepare compound 4a. The reaction components and analytical results were as specified: 3b (2.06 g, 5.0 mmol), Mes_2_BF (1.41 g, 5.27 mmol), *n*-butyllithium (2.5 M in hexanes, 6.0 mmol). Purification of the raw product *via* silica gel chromatography (PE/DCM, v/v = 5/1) yielded a yellow solid. Yield: 2.37 g (72%). ^1^H NMR (600 MHz, CDCl_3_): *δ* 7.46 (s, 2H, Ar*H*), 7.43 (d, 4H, *J* = 8.4 Hz, Ar*H*), 7.19 (d, 2H, *J* = 8.4 Hz, Ar*H*), 7.06 (d, 2H, *J* = 8.4 Hz, Ar*H*), 6.91 (d, 4H, *J* = 8.4 Hz, Ar*H*), 6.67 (s, 4H, Ar*H*), 3.82 (s, 6H, *O*C*H*_3_), 2.40 (s, 12H, C*H*_3_), 2.19 (s, 6H, C*H*_3_); ^13^C NMR (151 MHz, CDCl_3_): *δ* 159.26, 152.90, 142.21, 140.64, 138.05, 137.59, 137.17, 132.16, 129.92, 128.07, 127.82, 125.42, 125.03, 124.70, 114.20, 55.36, 23.93, 20.99. HRMS (APCI) *m*/*z*: [M + H]^+^ calcd for C_44_H_43_BNO_2_S, 660.3107, found, 660.3088. Anal. calcd for C_44_H_42_BNO_2_S: C, 80.11; H, 6.42; N, 2.12. Found: C, 80.06; H, 6.37; N, 2.06. Fluorescence emission (in solid) (*λ*_ex_ = 360 nm): *λ*_emis_ = 550 nm (*Φ* = 0.19; *τ* = 4.96 ns).

#### 10-(Dimesitylboraneyl)-3,7-bis(4-(trifluoromethyl)phenyl)-10*H*-phenothiazine (4c)

Compound 4c emerged through a synthetic route analogous to that employed for compound 4a. The reaction components and analytical results were as specified: 3c (2.44 g, 5.0 mmol), *n*-butyllithium (2.5 M in hexanes, 2.4 mL, 6.0 mmol), Mes_2_BF (1.41 g, 5.27 mmol). The resulting mixture underwent purification *via* silica gel chromatography (PE/DCM, v/v = 10/1), yielding a white solid. Yield: 2.83 g (77%). ^1^H NMR (600 MHz, CDCl_3_): *δ* 7.63 (d, 4H, *J* = 8.4 Hz, Ar*H*), 7.59 (d, 4H, *J* = 8.4 Hz, Ar*H*), 7.52 (d, 2H, *J* = 1.8 Hz, Ar*H*), 7.25 (d, 2H, *J* = 8.4 Hz, Ar*H*), 7.14 (dd, 2H, *J* = 8.4 Hz, *J* = 1.8 Hz, Ar*H*), 6.69 (s, 4H, Ar*H*), 2.41 (s, 12H, C*H*_3_), 2.20 (s, 6H, C*H*_3_); ^13^C NMR (151 MHz, CDCl_3_): *δ* 143.41, 142.98, 140.62, 137.50, 136.60, 130.13, 129.40, 128.17, 128.07, 127.03, 125.74, 125.70, 125.68, 125.47, 125.11, 123.31, 23.89, 21.00. HRMS (APCI) *m*/*z*: [M + H]^+^ calcd for C_43_H_37_BF_6_BNS, 736.2644, found, 736.2629. Anal. calcd for C_43_H_36_BF_6_NS: C, 71.84; H, 4.93; N, 1.90. Found: C, 71.76; H, 4.88; N, 1.84. Fluorescence emission (in solid) (*λ*_ex_ = 360 nm): *λ*_emis_ = 473 nm (*Φ* = 0.14; *τ* = 10.13 ns).

#### 10-Benzhydryl-3,7-di-*p*-tolyl-10*H*-phenothiazine (4d)

In an argon atmosphere, 3,7-di-*p*-tolyl-10*H*-phenothiazine (3a) (3.79 g, 10 mmol) was introduced into 100 mL of anhydrous THF at 0 °C, succeeded by portionwise incorporation of 0.6 g sodium hydride (15 mmol, 60% dispersion in mineral oil). The reaction solution was maintained under stirring at 0 °C for 30 min, after which brommodiphenylmethane (2.7 g, 11 mmol) was introduced. The mixture was allowed to warm to ambient conditions over 12 h. The reaction was terminated by adding distilled water (50 mL), succeeded by extraction utilizing DCM (3 × 50 mL). The pooled organic phases underwent drying over NaHCO_3_ and concentration, yielding a white solid. The target compound was procured *via* column chromatography, yield: 3.48 g (64%). ^1^H NMR (600 MHz, DMSO): 7.51–7.46 (m, 10H, Ar*H*), 7.33(t, 4H, *J* = 7.8 Hz, Ar*H*), 7.22–7.19 (m, 8H, Ar*H*), 6.75(s, 2H, Ar*H*), 6.74 (s, 1H, C*H*) 2.30 (s, 6H, C*H*_3_); ^13^C NMR (151 MHz, DMSO): *δ* 144.10, 144.08, 140.86, 136.89, 136.43, 136.29, 136.26, 134.99, 129.93, 129.07, 129.01, 128.39, 127.61, 127.23, 126.37, 125.15, 124.91, 124.89, 120.09, 67.33, 21.09. HRMS (APCI) *m*/*z*: [M + H]^+^ calcd for C_39_H_32_NS, 546.2255, found, 546.2273. Anal. calcd for C_39_H_31_NS: C, 85.83; H, 5.73; N, 2.57. Found: C, 85.78; H, 5.67; N, 2.51. Fluorescence emission (in THF) (*λ*_ex_ = 360 nm): *λ*_emis_ = 460 nm (*Φ* = 0.06; *τ* = 4.83 ns).

### Assembly of the electrochromic devices

The initial ITO glass plates measured 100.0 × 100.0 × 1.1 mm with 10 Ω resistance. The ITO glass plates underwent cutting processes using a glass cutter to accommodate various ECD specifications. A pair of ITO-coated glass pieces (5.0 cm × 2.0 cm) were secured together using 3 M double-side adhesive tapes (width: 4.0 mm, thickness: 0.5 mm), creating an internal cavity. The compounds 4a–4d underwent dissolution in DMF medium at 5 × 10^−3^ M concentration, using ionic liquid ethyl-3-methylimidazolium bis(trifluoromethylsulfonyl) as the electrolyte. Following the injection of this mixture into the cavity *via* syringe, the cavity underwent additional sealing utilizing UV-cured gasket material.

## Result and discussion

### Formation and analysis

**Scheme 1:**
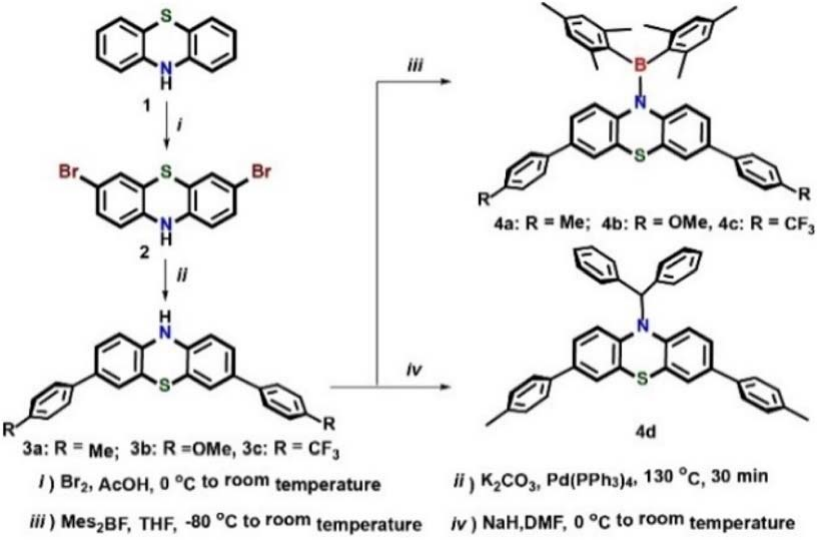
Synthetic routes of diarylboryl-phenothiazines (4a, 4b, 4c), and 4d.

The preparation of boryl derivatives (4a, 4b, and 4c) appears in Scheme 1. Precursors 3a, 3b, and 3c were generated through Suzuki–Miyaura cross-coupling, utilizing tetrakis(triphenylphosphine)palladium as the catalytic agent at 130 °C for 30 min. The target boryl derivatives were acquired through a one-step process by reacting precursors 3a, 3b, and 3c with *n*-butyllithium, succeeded by dimesitylfluoroborane incorporation, which captured the nitrogen anion of PTZ to generate boryl derivatives 4a, 4b, and 4c, delivering yields of 76%, 72%, and 77%, respectively. These boryl derivatives demonstrate stability against air and moisture both as solids and in solution. For comparative analysis of photophysical and electrochemical characteristics of the BN-containing boryl derivatives, a C–N-containing counterpart (4d) was produced as a white solid with 64% yield through the reaction of 3,7-di-*p*-tolyl-10*H*-PTZ (3a) with NaH and bromodiphenylmethane.

### Photophysical properties

**Fig. 1:**
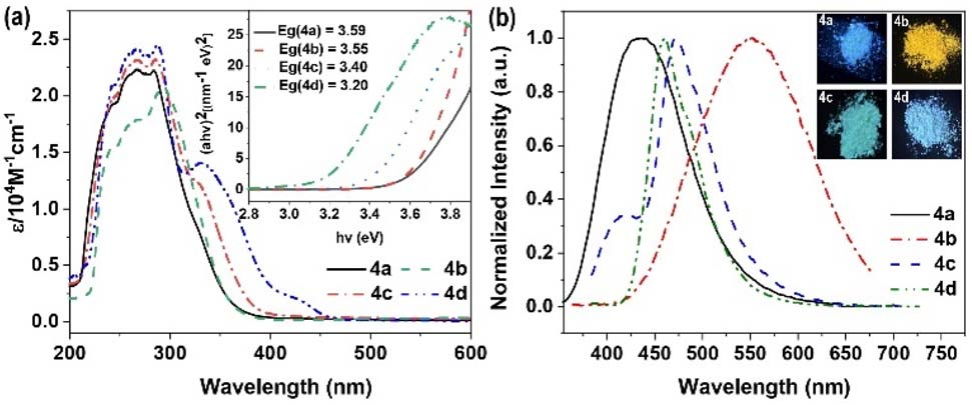
(a) UV-vis spectrum of 4a, 4b, 4c, and 4d in tetrahydrofuran solutions; calculated optical band gaps are shown as insets; (b) emission of materials in solid and insert picture: fluorescence color of the corresponding solids under UV light (365 nm).

Owing to their distinctive optical characteristics, boryl derivatives have attracted substantial interest in material applications.^39,42^ In contrast to the photophysical and electrochemical features of the 4d, compounds 4a, 4b, and 4c exhibit notable distinguishing properties. The UV-vis profiles of the BN-containing derivatives along with CN-containing compound 4d are depicted in Fig. 1a. All compounds (4a, 4b, 4c, and 4d) demonstrate comparable absorption profiles, exhibiting substantial visible-region absorption. The UV-vis spectra in THF display narrow bands with absorption peaks (*λ*_max_) at 284 nm, 293 nm, 286 nm, and 287 nm, respectively, attributable to π–π* transitions. Compound 4d displays a minor absorption band at 331 nm, corresponding to the forbidden n–π* transition. The optical band gaps (*E*_g_) determined from the absorption onset wavelengths in solution for 4a, 4b, 4c, and 4d yielded values of 3.59 eV, 3.55 eV, 3.40 eV, and 3.20 eV, respectively.

The photoluminescence properties of these boryl derivatives underwent examination. In tetrahydrofuran solution, compound 4a displays minimal luminescence, yet demonstrates amplified emission characterized by a 434 nm peak, displaying a fluorescence quantum yield (*Φ*_F_) of 0.16 and a fluorescence lifetime measuring 5.92 ns (Fig. 1b, S1a and S2a†). Analogous behaviors are observed in the methoxy- and trifluoromethyl-functionalized derivatives, 4b and 4c, exhibiting limited emission in THF solution while demonstrating heightened fluorescence in solid form (4b, *λ*_max,em_ = 550 nm, *Φ* = 0.19, *τ* = 4.96 ns; 4c, *λ*_max,em_ = 473 nm, *Φ* = 0.14, *τ* = 10.13 ns) (Fig. S1b, c, S2b and c†). Distinctively, compound 4d manifests luminescence in both solid state and tetrahydrofuran solution (*λ*_max,em_ = 460 nm, *Φ* = 0.06, *τ* = 4.83 ns) (Fig. S1d and S2d†). The data indicates that the B–N moiety substantially influences the optical characteristics, displaying a distinctive AIE behavior with weak fluorescence in THF solution yet enhanced emission intensity in the solid form.^37,43^

**Fig. 2:**
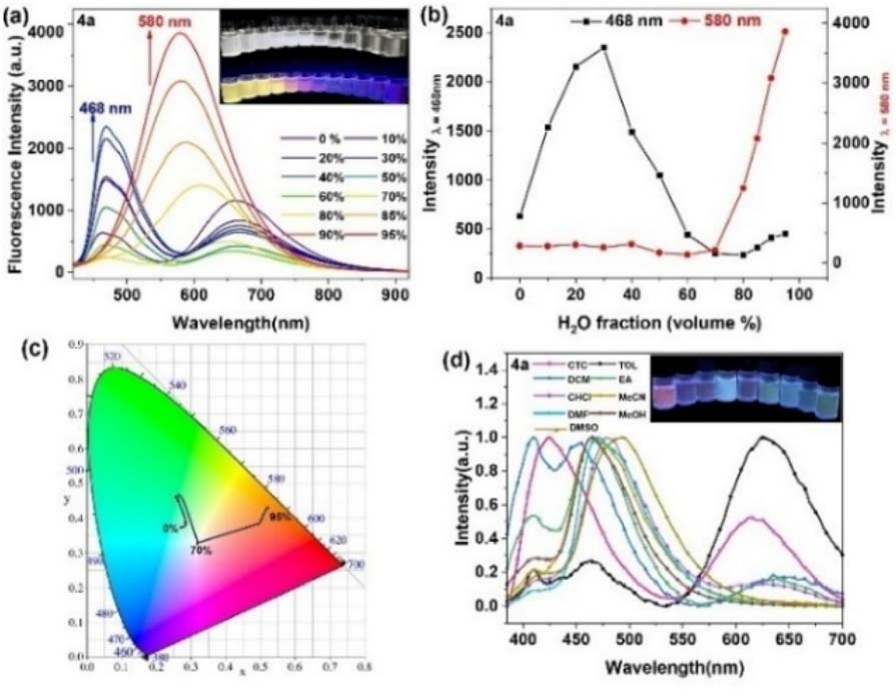
(a) PL spectra of 4a in THF/H_2_O mixtures with different ratios (insets: pictures of the fluorescence from 0% to 95%, *λ*_ex_ = 365 nm); (b) the fluorescence intensity of 4a under the different ratios of THF/water; (c) CIE chromaticity. (d) PL spectra of 4a in different solutions.

To understand the AIE characteristics of diarylboryl–PTZ, an examination of their aggregate formation was executed in mixed solvent condition (Fig. 2). The evaluation of AIE performance took place through the addition of H_2_O (poor solvent) into a THF solution (good solvent) of diarylboryl–PTZ. Compound 4a displayed minimal emission in THF solution, yet when H_2_O content surpassed 30 vol%, the fluorescence transformed progressively from faint luminescence to intense blue, showing enhanced emission at 468 nm (CIE chromaticity coordinates, *x* = 0.2601, *y* = 0.4494). After H_2_O concentration surpassed 95%, the initial emission peak at 468 nm disappeared entirely, while a fresh peak emerged at 580 nm, transforming the emission from blue to yellow (CIE chromaticity coordinates, *x* = 0.5248, *y* = 0.4308), resulting from the aggregate-state fluorescence of diarylboryl–PTZ derivatives (Fig. 2). Compounds 4b and 4c demonstrated comparable characteristics, showing minimal luminescence in solution but increased fluorescence intensity during aggregation (Fig. S10 and S11†). In comparison, the structurally analogous diarylboryl–PTZ compound 4d demonstrated strong fluorescence in THF solution at 461 nm, but its fluorescence decreased as the water fraction increased (Fig. S12†). The unique fluorescence behaviors of diarylboryl–PTZ and 4d to water stem from the combined effects of solvent polarity and ICT states.^44^ This difference in luminescence behavior necessitated additional examination of the mechanism underlying the AIE effect of diarylboryl–PTZ. From a structural standpoint, the diarylboryl–PTZ derivatives incorporate D (PTZ) and A (diarylboryl) units, and these D–A molecules frequently display solvatochromic characteristics. The solvatochromic behavior of diarylboryl–PTZ finds an explanation through the twisted ICT (TICT) mechanism.^42,45^

**Fig. 3:**
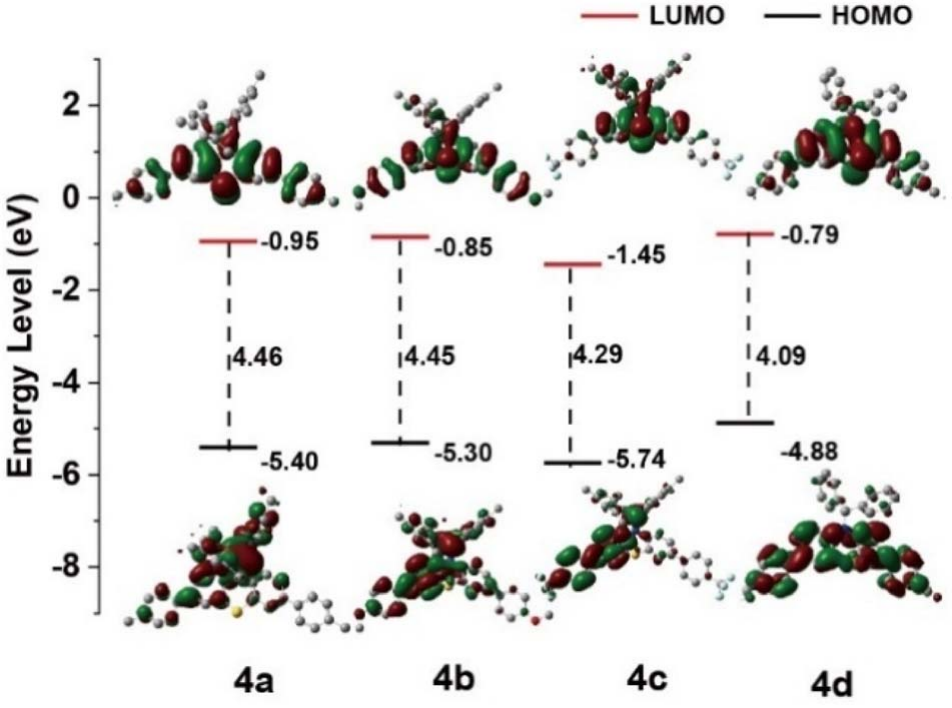
Calculated the HOMO and LUMO spatial electron distributions of 4a, 4b, 4c and 4d.

To explore the fundamental AIE mechanism within these compounds, investigations of photophysical properties across solvents exhibiting varying polarities were conducted to assess the presence of the TICT effect. The 4a, 4b, and 4c fluorescence signals displayed orange emission peaks at 630 nm, 627 nm, and 591 nm, respectively, when measured in low-polarity solvents, including phenixin and toluene. Alternatively, measurements in high-polarity solvents like DMF, methanol, and DMSO revealed diminished luminescence between 410 nm and 450 nm. The observed blue shift and luminescence reduction with elevated solvent polarity suggested that the intramolecular CT excited-state possessed greater dipolar characteristics compared to the ground state, attributed to charge redistribution during Franck–Condon excited state relaxation, rather than direct ground state transitions (Fig. S13 and Table S8, ESI†).^46,47^ The HOMO and LUMO electron density distribution patterns indicated a π–π* transition featuring charge transfer characteristics (Fig. 3), corresponding with experimental observations. Compound 4d demonstrated distinct behavior, exhibiting blue emission ranging from 452 nm to 464 nm across various solvent polarities (Fig. S12d†).

The CV analysis in DCM solution was utilized to investigate the electrochemical characteristics of compounds 4a, 4b, 4c, and 4d (Fig. S14, ESI†), with data compiled in Table S9, ESI.† The voltammetric analysis revealed that 4a, 4b, and 4c demonstrated dual reversible oxidation signals at respective potentials of 0.79 V (*E*_HOMO_ = −5.59 eV), 0.86 V (*E*_HOMO_ = −5.66 eV), and 0.96 V (*E*_HOMO_ = −5.76 eV) *versus* Fc/Fc^+^ (Fig. 3 and S15–S18, ESI†). The corresponding LUMO energy levels, determined by subtracting optical band gap energies (*E*_g_) (4a, 3.59 eV; 4b, 3.55 eV; and 4c, 3.40 eV) from HOMO levels, yielded measurements of −2.00, −2.11, and −2.36 eV, respectively. Under identical experimental conditions, compound 4d exhibited oxidation at 0.78 V, corresponding to elevated HOMO (*E*_HOMO_ = −5.58 eV) and LUMO (*E*_LUMO_ = −2.38 eV) energy states, aligning with computational predictions. The distinct redox behavior, modulated by B and N atomic orbital contributions in diarylboryl–PTZ structures, results in modified electronic characteristics, with 4a, 4b, and 4c displaying reduced HOMO and LUMO levels *versus*4d, enhancing their electron transport capabilities.^48,49^

### Ratiometric fluoride-ion sensing properties

**Fig. 4:**
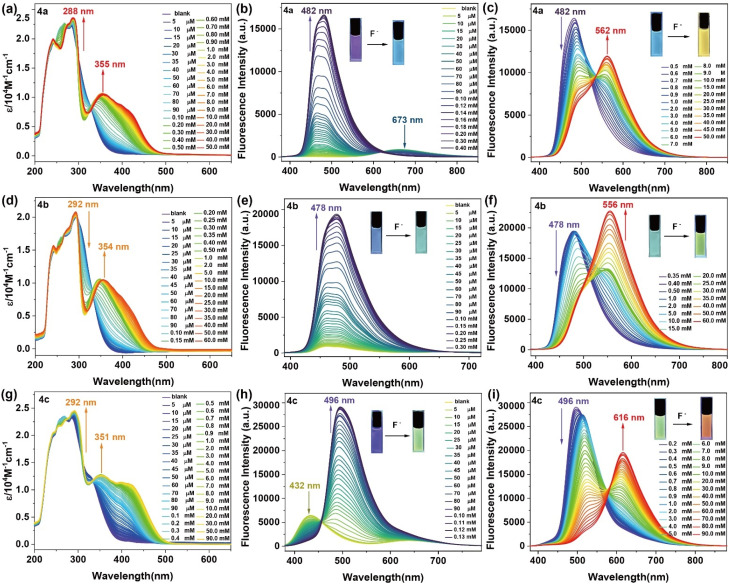
(a), (d) and (g) The UV/vis absorption spectral changes of diarylboryl–PTZ (4a, 4b, and 4c) after addition of F^−^ in the different concentrations; (b), (c), (e), (f), (h) and (i) the fluorescence emission spectra of after addition of F^−^ (insets: photographs of the fluorescence after added F^−^, *λ*_ex_ = 365 nm).

Given their inherent Lewis acidic nature,^50^ three-coordinate organoboranes can accept electron pairs from Lewis bases, specifically fluoride, generating fluoroborate anions. Such interactions are conventionally interpreted as addition processes, wherein the fluoride anion transfers its electron pair into the vacant p-orbital at the boron atom, establishing a robust B–F bond that interrupts the extended π-conjugation, subsequently modifying the photophysical characteristics.^51^ Following this principle, three-coordinated organoboranes demonstrate selective and sensitive fluoride anion recognition. To evaluate the fluoride-detection capabilities of diarylboryl–PTZ, absorption and emission titration analyses were performed in THF medium utilizing tetrabutylammonium fluoride as the fluoride donor (Fig. 4). Sequential introduction of fluoride into a 4a solution produced a marked reduction in the primary absorption signal at 288 nm, coupled with a bathochromic shift to 355 nm. Similarly, 4b and 4c displayed analogous responses, with absorption signals progressively shifting from 292 nm toward 354 nm and 351 nm, respectively, upon increasing fluoride concentrations. These experimental observations aligned with computational predictions (Fig. S7–S9 and Tables S5–S7, ESI†). Fluorescence spectral alterations of diarylboryl–PTZ were examined in THF medium (Fig. 3b and c). While 4a displayed minimal luminescence initially, its emission signal at 482 nm intensified progressively with fluoride ions (F^−^) addition up to 0.4 mM, transitioning from faint to intense blue emission, demonstrating fluoride-activated sensing behavior. Subsequent excess F^−^ addition (reaching 60 mM) decreased the 482 nm emission while generating a signal at 562 nm. The fluoride binding manifested visually through a color transition from blue to yellow. Compounds 4b and 4c exhibited parallel behavior, with emission signals at 478 nm (4b) and 496 nm (4c) intensifying upon F^−^ addition until 0.13 mM and 0.3 mM, respectively. Additional F^−^ introduction up to 60 mM (4b) and 90 mM (4c) generated new signals between 556 nm and 616 nm, accompanied by blue-to-yellow emission transitions. Detection threshold values for 4a, 4b, and 4c, determined *via* triple standard deviation methodology, measured 4.33 μM, 15.60 μM, and 10.47 μM, respectively (Fig. S19 and ESI†). Notably, 4d remained unresponsive to fluoride addition under identical experimental conditions. These observations indicate that fluoride coordination transforms the boron center from three- to four-coordinate geometry, converting its electronic character from acceptor to donor.^52^

**Fig. 5:**
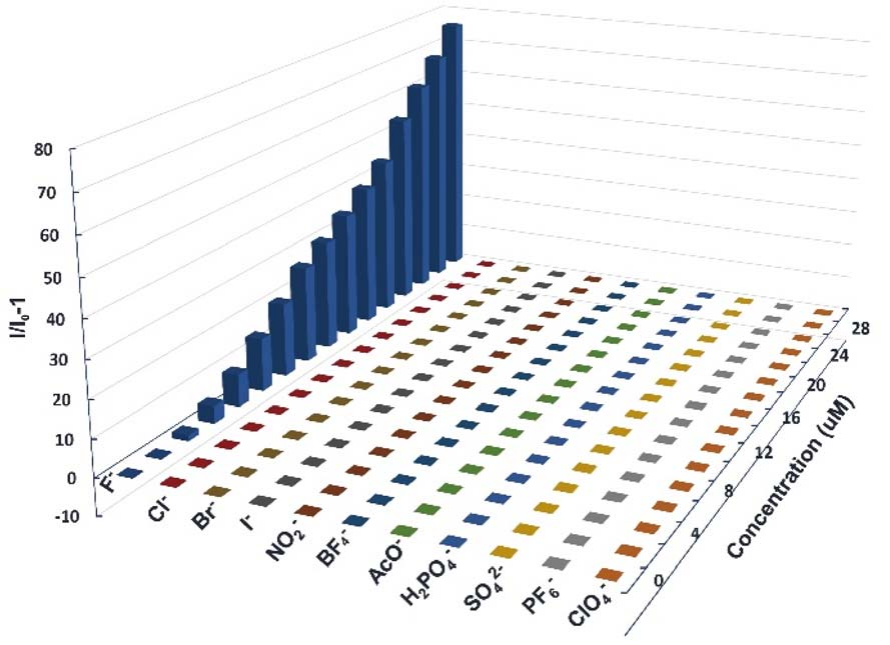
The fluorescence emission of 4a at different concentrations of anions (10 μM, *λ*_ex_ = 365 nm).

The anion-detection specificity of the diarylboryl–PTZ derivatives underwent evaluation through the concurrent addition of different anions encompassing F^−^, I^−^, Cl^−^, Br^−^, ClO_4_^−^, NO_3_^−^, BF_4_^−^, AcO^2−^, PF_6_^−^, H_2_PO_4_^−^ and SO_4_^2−^. The graphical representation of (*I*/*I*_0_ − 1) *versus* concentrations of F^−^ and alternative anions demonstrated significant variations in both intensity and emission wavelength for 4a when introducing fluoride ions, whereas other anions produced negligible alterations in the fluorescence spectra (Fig. 5).

**Fig. 6:**
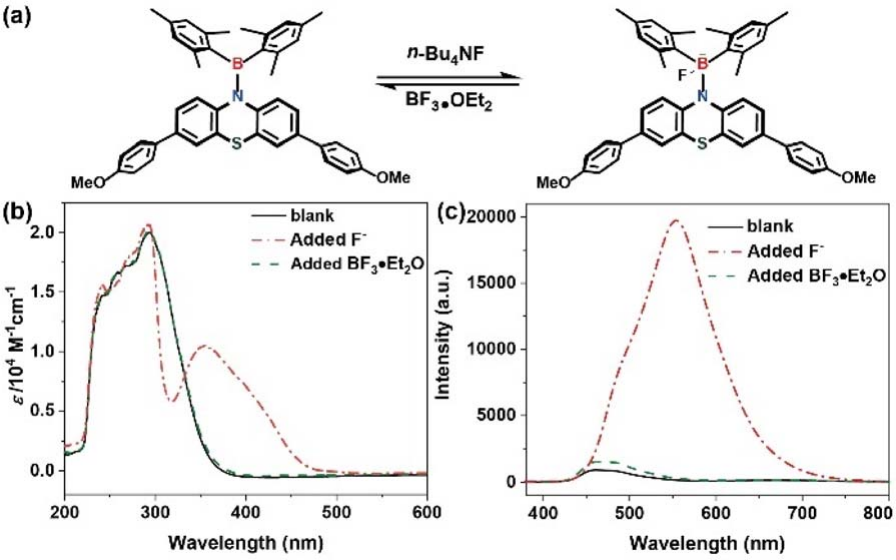
(a) Proposed mechanism of 4b with F^−^; (b) UV-vis spectra of 4b; (c) fluorescence emission spectra of 4b (solid line: THF solution; dashed dotted line: added F^−^; dashed line: added BF_3_·OEt_2_).

The electron-depleted boron atoms within diarylboryl–PTZ moieties undergo coordination upon F^−^ addition, transforming from trigonal to tetrahedral geometry. Additional studies examining fluoride-boron interactions employed reversibility tests utilizing UV-vis and fluorescence spectroscopic methods. Subsequently, the introduction of the F^−^ scavenging agent BF_3_·OEt_2_ to the THF solution resulted in near-complete restoration of the tetrahedral boron complex (4b–F) spectra to initial conditions (Fig. 6). This observation indicates that diarylboryl–PTZ maintains structural integrity without degradation following fluoride coordination. Such findings demonstrate concordance with DT-DFT computational analysis (Gaussian 09, B3LYP, 6-31g*) (Fig. S7, ESI†), supporting the experimental data.

### Fabrication of electrochromic devices (ECDs) and spectroelectrochemical properties

**Fig. 7:**
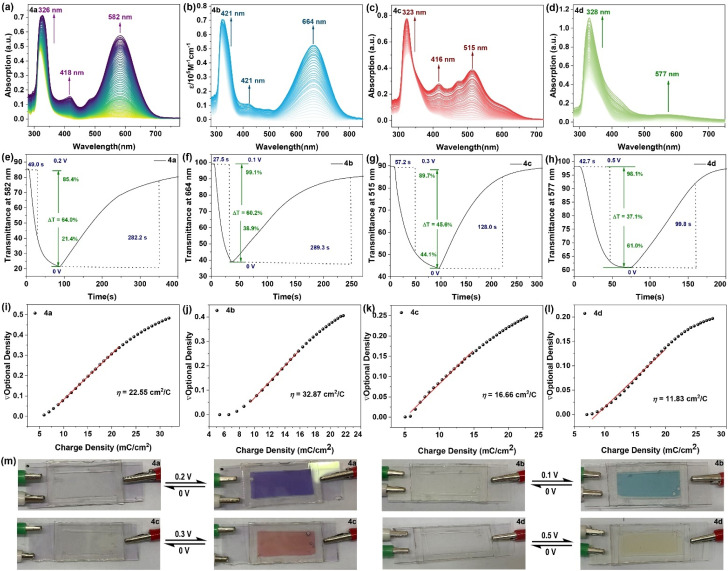
Spectroelectrochemistryin DMF solution (a) 4a, potential: 0 to 0.2 V; (b) 4b, potential: 0 to 0.1 V; (c) 4c, potential: 0 to 0.3 V; (d) 4d, potential: 0 to 0.5 V; (e–h) transmittance changes of the device under different voltages; (e–l) calculated color efficiency (*η*) of ECDs by the plots of Δ*D versus* injected Δ*Q*; (m) the color change of ECDs in the different potentials.

Phenothiazine (PTZ) exhibits a unique nonplanar ring with a butterfly conformation, facilitated by powerful electron donors like nitrogen and sulfur heteroatoms. This structure is characterized by a lower oxidation potential and easy to produce stable radical cations, making it suitable for applications in electrochromic devices when subjected to specific voltage stimuli. To explore the effects of voltage stimulation on these PTZ derivatives, proof-of-concept electrochromic devices were constructed. Indium–tin oxide (ITO)-coated glasses, with their excellent transmittance and conductivity, were used as electrodes, allowing for easy observation of the color changes induced by voltage. The device performance, including observed color changes, coloration efficiency, spectroelectrochemical data, and transmittance changes, are shown in Fig. 7. In its original state at 0 V, the device containing 4a in DMF solution is colorless and transparent, with the molecular structure in its neutral state and UV-vis absorption maxima at 326 nm. Upon applying a gradually increasing voltage from 0 V to 0.2 V, the absorption intensity at 326 nm increased, and two new absorption bands appeared at 418 nm and 582 nm, corresponding to the formation of radical cation species generated during the electrochemical oxidation process (Fig. 7a). The device color changed from colorless to dark violet (Fig. 7m). Similarly, the devices containing 4b and 4c showed a colorless appearance at 0 V, with absorption intensities increasing at 421 nm and 664 nm (4b) and 416 nm and 515 nm (4c) after applying specific voltages (4b: 0.1 V, 4c: 0.3 V), resulting in color changes to aqua green (4b) and brown (4c) (Fig. 7b and c). In contrast, compound 4d did not show significant changes in absorption spectrum or device color upon voltage application (Fig. 7d).

The transmittance of the electrochromic devices (ECDs) is a critical index for evaluating device performance. The response time was measured as the response time required to reach 90% of the transmittance change from the original state to the maximum coloring state in the absorption wavelength. Fig. 7e shows the optical transmittance intensity of 4a at 582 nm over time under 0.2 V and 0 V. The coloration time was 49.0 s, and it returned to the neutral state in 282.2 s. The optical contrast (Δ*T*%) of 4a was calculated to be 64.0%, derived from the coloration state (21.4%) and the neutral colorless state (85.4%).^53^ The transmittance of the ECDs made from 4b, 4c, and 4d was also measured, with results shown in Fig. 7b–d. The coloration times and neutral colorless states at 664 nm (4b), 515 nm (4c), and 577 nm (4d) were 27.5 s and 289.3 s for 4b, 57.2 s and 128.0 s for 4c, and 99.8 s for 4d. Corresponding color contrast ratios of the ECDs were 60.2% (4b), 45.6% (4c), and 37.1% (4d), respectively. Coloration efficiency (*η*), a key parameter for evaluating the performance of electrochromic devices (ECDs), is calculated using the slope of a linear fit of optical density (Δ*D*) *versus* charge density (Δ*Q*) plots. The equation for coloration efficiency is expressed as follows:
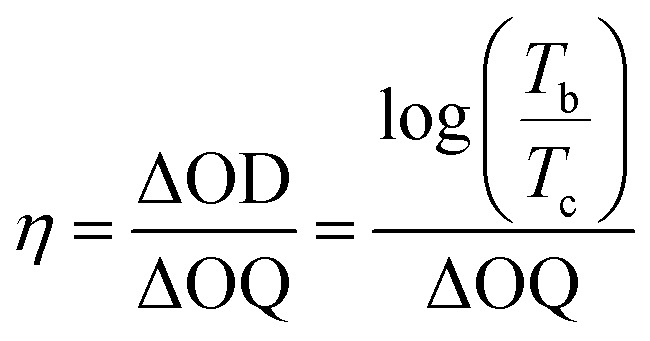
where the change in optical density (ΔOD) is obtained by using the ratio of transmittance in the coloration state (*T*_b_) to transmittance in the bleached state (*T*_c_), and Δ*Q* represents the charge density of the device, which calculate by the integral calculus *via* electric current (*I*) with color change time (*t*).^54,55^ The estimated coloration efficiency of ECDs containing 4a, 4b, 4c, and 4d were calculated to be 22.55 cm^2^ C^−1^, 32.87 cm^2^ C^−1^, 16.66 cm^2^ C^−1^, and 11.83 cm^2^ C^−1^, respectively (Fig. 7i–l).

### Mechanofluorochromic (MFC) properties

**Fig. 8:**
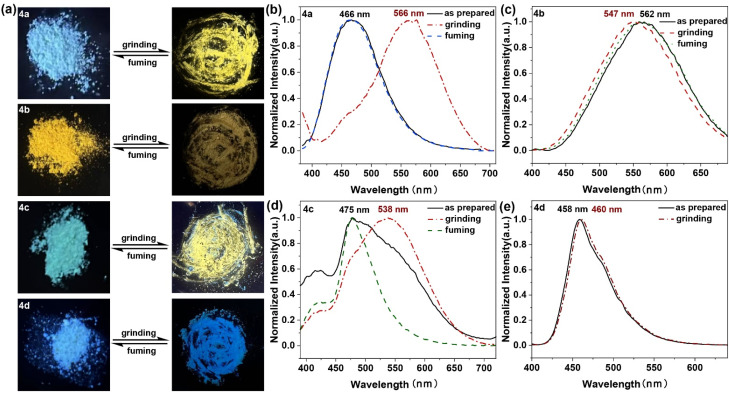
(a) Fluorescence color changes under grinding and wetting, *λ*_ex_ = 365 nm. (b–e) Normalized fluorescence of solids (solid line: as prepared; dashed dotted line: under grinding; dashed line: fuming).

Most of the AIE molecules, which include rotatable aryl groups, exhibit intermolecular loose packing and weak π–π interactions in the aggregated state, making them central to the development of MFC (mechanically flexible and color-changing) materials. With the highly twisted molecular skeletons and the prominent ICT characteristics of AIE, it is hypothesized that diarylboryl–PTZ may exhibit controllable fluorescence color change stimulated by external forces. To test this hypothesis, the MFC properties of 4a, 4b, 4c, and 4d were explored under various conditions (preparation, grinding, and fuming). The color of fluorescence changes of the diarylboryl–PTZ powders to grinding and solvent-fuming processes are shown in Fig. 8. Under UV excitation (365 nm), the original crystalline powders of 4a emitted a pale blue light with a maximum emission at 466 nm. However, after grinding briefly in a mortar or on a quartz substrate using a pestle, the powder color shifted from pale blue to bright yellow, with the emission maximum red-shifting to 566 nm, a 100 nm shift compared to the as-synthesized state (Fig. 8b). Notably, exposing the sample to DCM vapor for 5 minutes at room temperature caused the maximum emission wavelength of 4a to blue-shift back to its original value, similar to the as-prepared solid. The emission spectra remained consistent after the first grinding and re-grinding, and the process of grinding-fuming was repeated up to six times without fatigue (Fig. S20a and b, ESI†), demonstrating the good reversibility of the MFC performance. which fully reflected the good reversibility of the MFC performance. Fig. S20c† illustrates the XRD patterns of 4a solid powders under three distinct conditions: as-prepared, ground, and wetted states. The as-prepared powder exhibited multiple sharp, distinct diffraction peaks, demonstrating an ordered crystal structure. In contrast, the ground powder of 4a revealed broad and indistinct diffractograms, indicating the disruption of the ordered crystalline arrangement through mechanical forces, resulting in significant amorphous phases. Notably, when the ground powders underwent exposure to DCM vapor, the XRD pattern displayed sharp and pronounced peaks comparable to the as-prepared powder, indicating the restoration of crystallinity through molecular reorganization. In contrast, compound 4b exhibited entirely different behavior; while the as-synthesized powder showed yellow emission at 562 nm, the emission exhibited a slight blue shift to 547 nm after grinding. Compound 4c showed properties similar to those of 4a, where the blue-emitting solid, under 365 nm UV light, displayed a maximum emission at 475 nm in the as-synthesized powder. When the blue-emitting powder was ground with a pestle, a bright yellow-emitting state was observed, and the fluorescence emission experienced a significant red-shift of 63 nm, from 475 nm to 538 nm. After exposing the ground powder of 4c to DCM vapor for 5 minutes, the original blue emission at 475 nm was recovered, and this process could be repeated several times. In contrast to the MFC behavior of diarylboryl–PTZ, compound 4d showed no significant change under external stimulation, including in the fluorescence color of the as-synthesized powder and its fluorescence emission. This difference in behaviour between diarylboryl–PTZ and the C–N-containing compound 4d can be attributed to the more twisted molecular structures in the former, resulting from the overlap of the empty p-orbital of the tricoordinate boron atom with an adjacent organic π-system.^56–58^

## Conclusions

In conclusion, several innovative donor–acceptor luminophores, specifically aminoboranes incorporating phenothiazyl groups, were fabricated and characterized. Analysis demonstrated that aminoborane derivatives 4a, 4b, and 4c displayed improved stability and reduced HOMO energy levels in comparison to the CN-functionalized compound 4d. The luminescent characteristics emerged from the combined effects of AIE, which charge transfer between the electron-abundant PTZ segment and electron-deficient boron (B) *via* the TICT mechanism. Based on their distinctive photophysical attributes, these diarylboryl–PTZ structures proved effective as “turn-on” fluorescent probes for fluoride recognition, exhibiting remarkable sensitivity and selectivity. Meanwhile, the diarylboryl–PTZ also exhibited reversible electrochromic properties under different voltages, with the color of the devices changing from colorless to dark violet, aqua green, and brown, respectively. Additionally, the emitting color of diarylboryl–PTZ changed from blue to yellow after grinding, and the emission maxima exhibited restoration when the ground samples were exposed to DCM vapor. These findings contribute markedly to understanding the development of novel fluorescent materials that respond to multiple stimuli and the fabrication of high-performance fluorescence chemosensors, mechanofluorochromic, and electrochromic devices.

## Data availability

The data presented in this article are available on request from the corresponding author.

## Conflicts of interest

There are no conflicts to declare.

## Supplementary Material

RA-015-D5RA01331C-s001
