# Supplementary material for: Multi-stimuli-responsive aggregation-induced emission of boryl substituted phenothiazine
Source: RSC Adv. 2025 May 12;15(20):15480–9. doi: 10.1039/d5ra01331c (PMC12067193; doi:10.1039/d5ra01331c)
Supplement: RA-015-D5RA01331C-s001 [file RA-015-D5RA01331C-s001.pdf]

Supplementary Information (SI) for RSC Advances.
This journal is © The Royal Society of Chemistry 2025

Supporting Information

for

Multi-stimuli-responsive aggregation-induced emission of boryl substituted phenothiazine

Guoqiang Li,‡[a] Yan Wang,‡[a] Yaohui Li,[a] Zengheng Wen,[a] Zhuang Luo,[a] Weijun Song,[a] and Weidong Zhang*[a]*

[a] School of Chemical Engineering, Qinghai University, Xining, 810016, China

E-mail: *zhangwd@qhu.edu.cn; songweijun1980@163.com*

Contents

1. The excitation and emission spectra 3

2. The fluorescence lifetime decay spectra 3

3. The Experimental and the Calculated UV-Vis spectra 4

4. Aggregation-induced fluorescence properties 15

5. Electrochemical properties 19

6. Comparison of HOMO/LUMO plots 20

7. Determination of the detection limit 23

8. Reversibility of the MFC performance 23

9. ^{1}H NMR and ^{13}C NMR spectra 24

10. HRMS spectra 28

11. Reference 30

1. The excitation and emission spectra

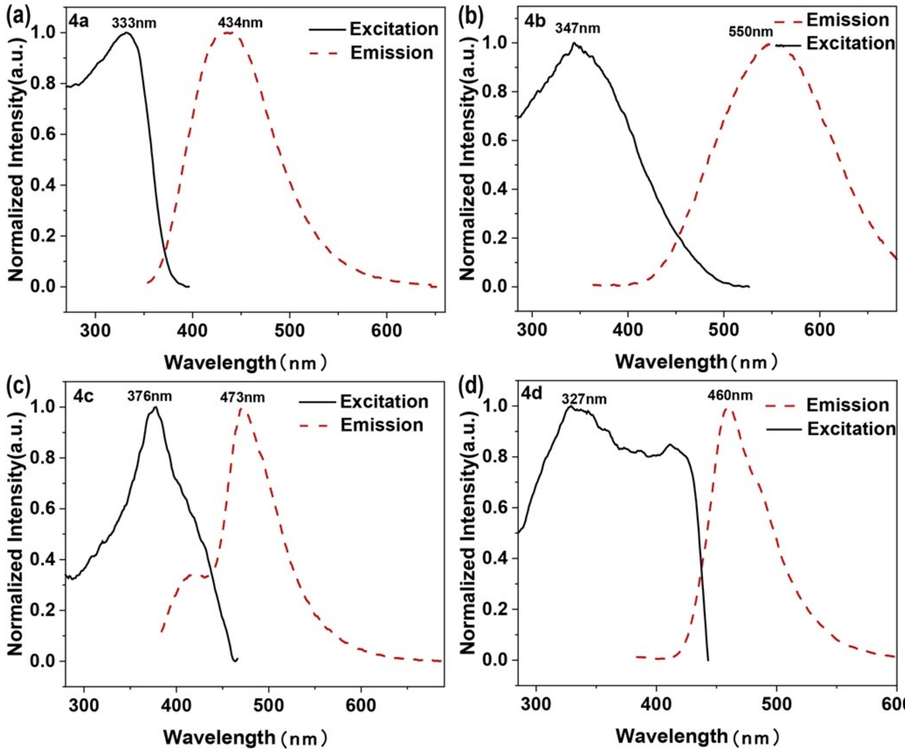

Figure S1. Fluorescence excitation (solid line) and emission spectra (dashed line).

2. The **fluorescence lifetime decay spectra**

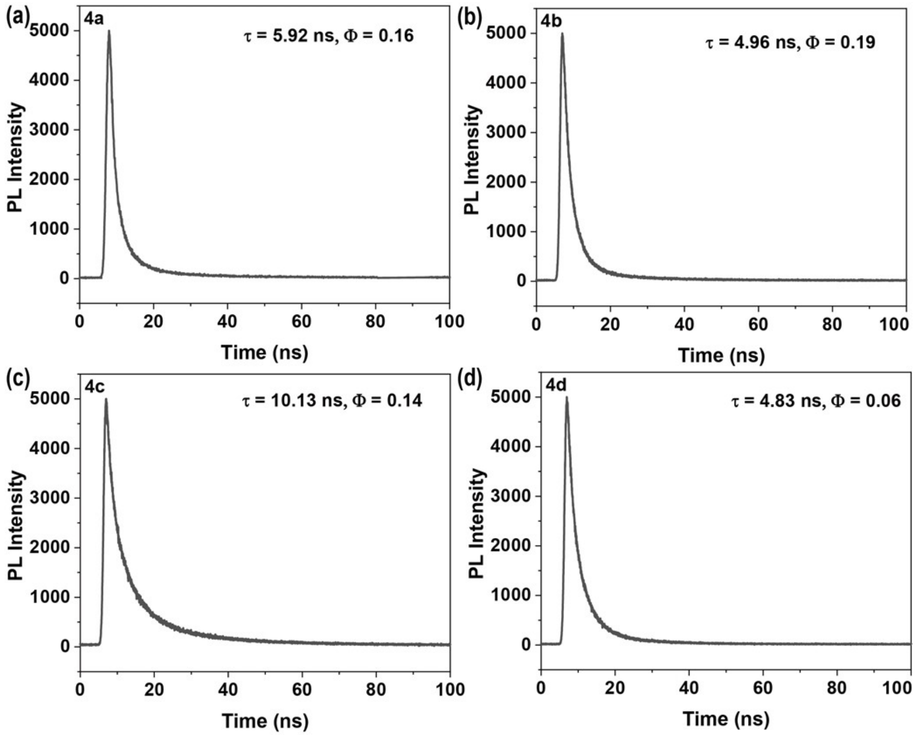

Figure S2. Fluorescence lifetime decay spectra of **4a-4d**.

3. The Experimental and the Calculated UV-Vis spectra

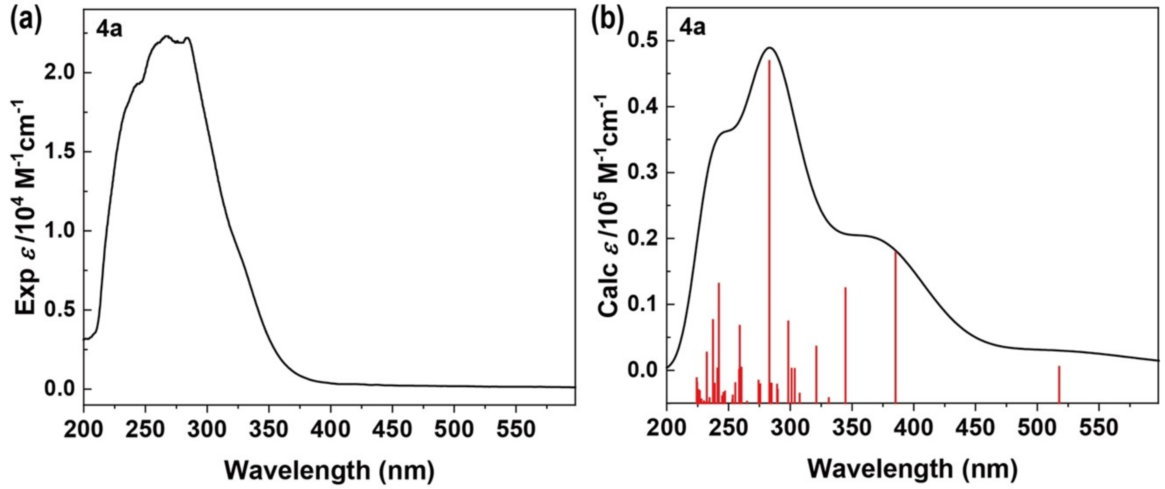

Figure S3. Computed, at the TD-DFT, B3LYP-D3/6-31g(d),[1-4] level of theory and experimental UV-vis spectra of **4a**.

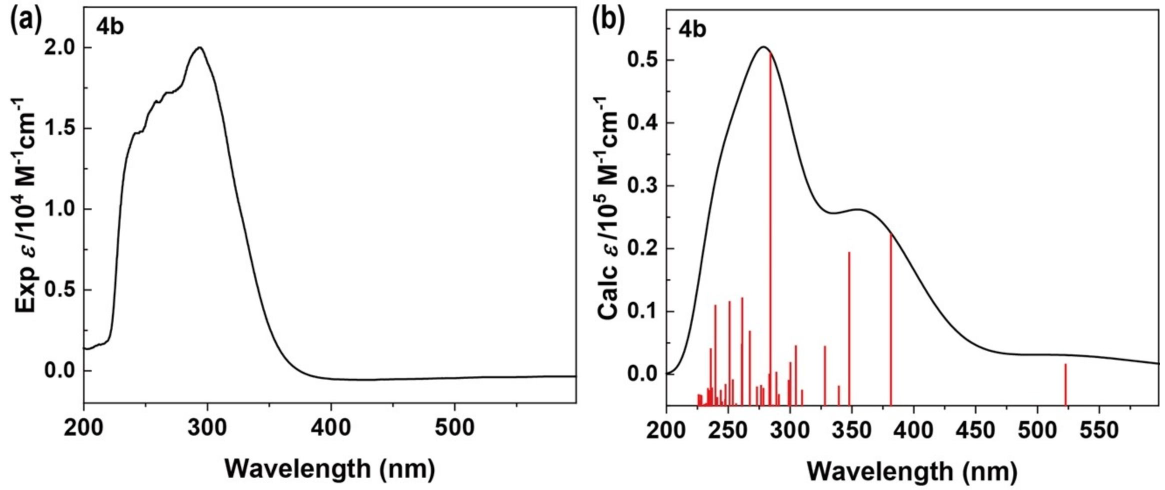

Figure S4. Computed, at the TD-DFT, B3LYP-D3/6-31g(d), level of theory and experimental UV-vis spectra of **4b**.

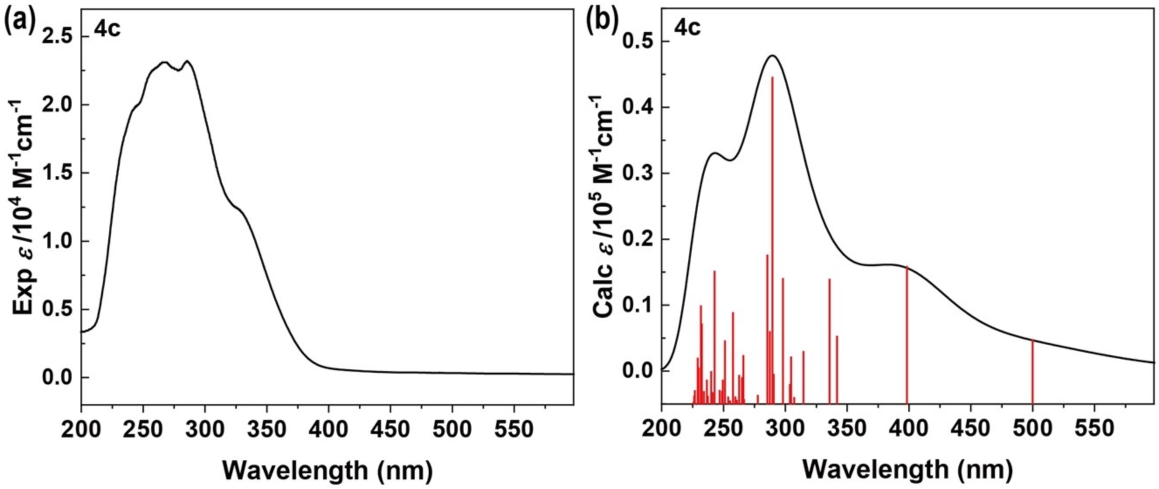

Figure S5. Computed, at the TD-DFT, B3LYP-D3/6-31g(d), level of theory and experimental UV-vis spectra of **4c**.

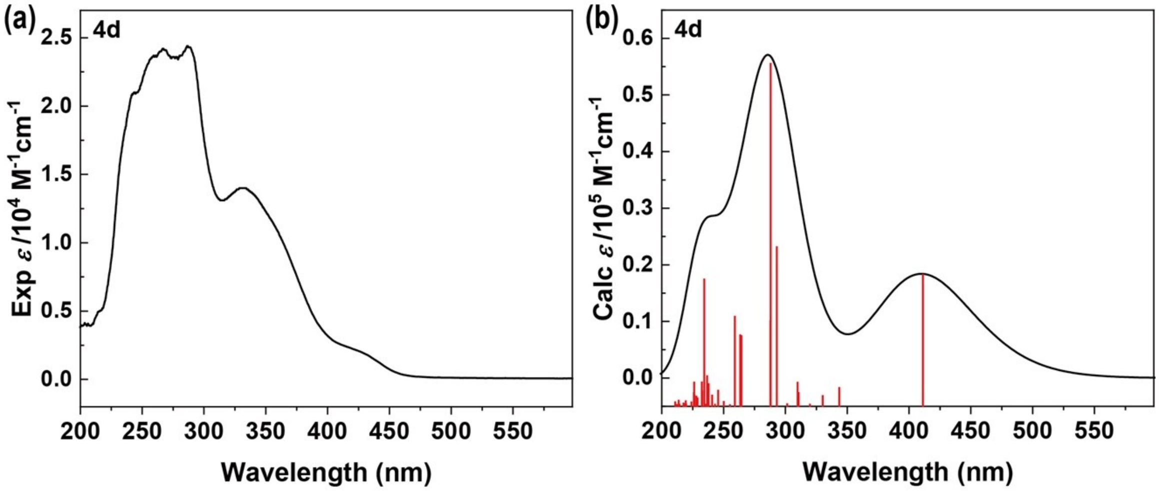

Figure S6. Computed, at the TD-DFT, B3LYP-D3/6-31g(d), level of theory and experimental UV-vis spectra of **4d**.

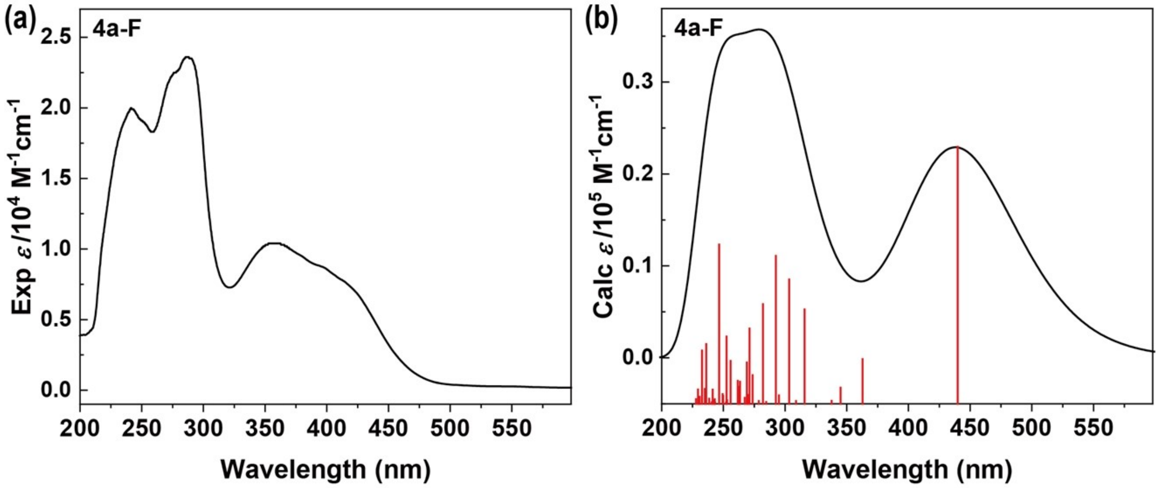

Figure S7. Computed, at the TD-DFT, B3LYP-D3/6-31g(d), level of theory and experimental UV-vis spectra of **4a-F**.

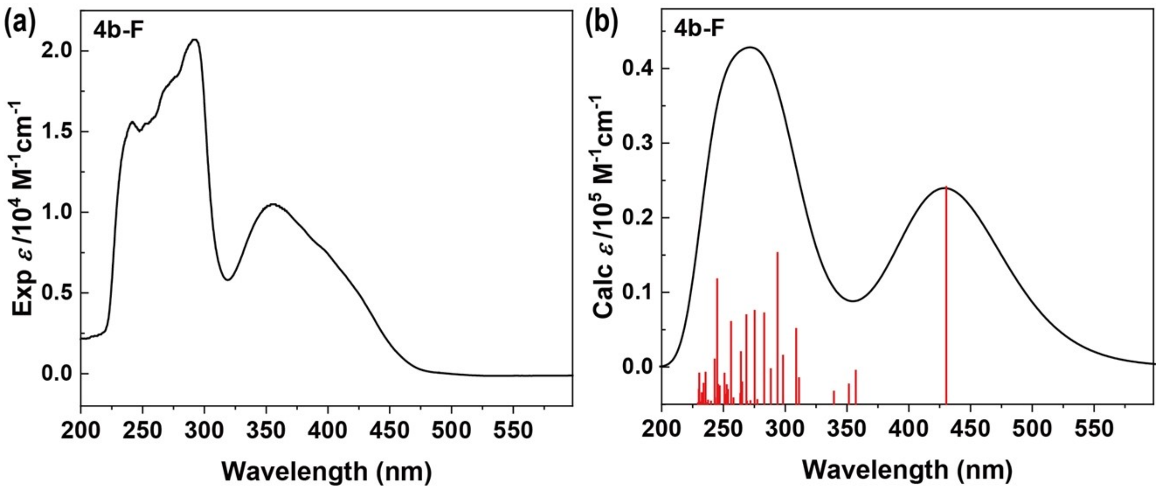

Figure S8. Computed, at the TD-DFT, B3LYP-D3/6-31g(d), level of theory and experimental UV-vis spectra of **4b-F**.

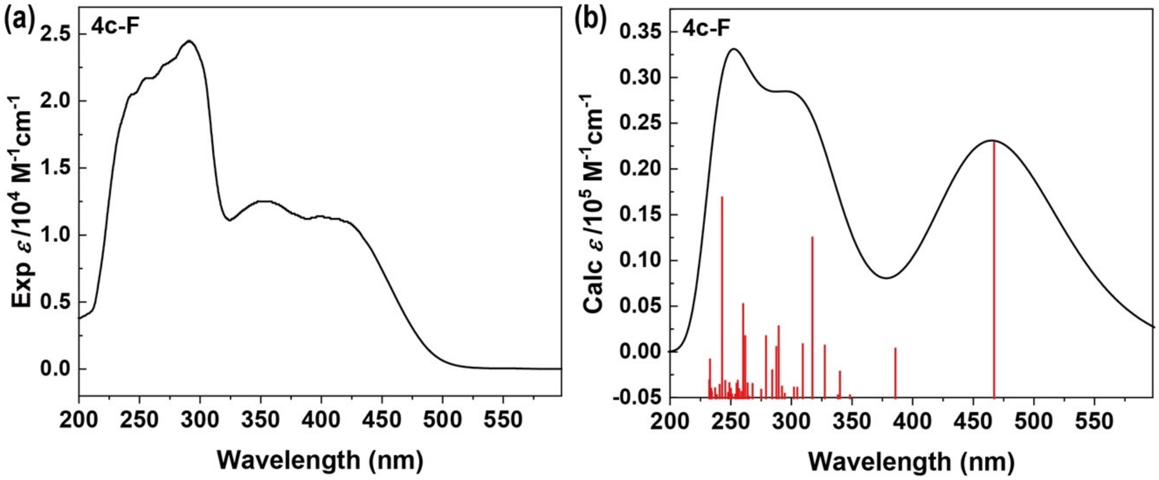

Figure S9. Computed, at the TD-DFT, B3LYP-D3/6-31g(d), level of theory and experimental UV-vis spectra of **4c-F**.

Table S1. Calculated ($\lambda_{TD\text{-}DFT}$) wavelengths of **4a**. Molecular orbitals (MOs) involved in the main electronic transition，f corresponds to the oscillator strength. (TD-DFT, B3LYP-D3/6-31g(d))

$\lambda_{TD\text{-}DFT}$	Oscillator Strength, f	MOs	
384.96	0.3482	HOMO-1 ->LOMO	16.36%
		HOMO ->LOMO+1	67.91%
344.42	0.2178	HOMO-1 ->LOMO	65.91%
		HOMO ->LOMO+1	15.95%
		HOMO ->LOMO+2	12.71%
320.75	0.1074	HOMO-3 ->LOMO	18.44%
		HOMO-2 ->LOMO	59.67%
		HOMO ->LOMO+2	27.34%
		HOMO ->LOMO+3	12.03%
298.02	0.1548	HOMO-5 ->LOMO	29.71%
		HOMO-4 ->LOMO	49.53%
		HOMO-3 ->LOMO	23.97%
		HOMO-2 ->LOMO	12.51%
		HOMO ->LOMO+3	19.24%
		HOMO ->LOMO+4	12.59%
258.87	0.1467	HOMO-10 ->LOMO	11.86%
		HOMO-8 ->LOMO	27.38%
		HOMO-2 ->LOMO+1	18.00%

		HOMO ->LOMO+8	15.67%
		HOMO ->LOMO+9	14.88%
		HOMO ->LOMO+10	51.02%
241.95	0.2265	HOMO-8 ->LOMO	10.78%
		HOMO-2 ->LOMO+2	61.34%
		HOMO-2 ->LOMO+3	15.89%
		HOMO-1 ->LOMO+3	16.75%
232.10	0.0958	HOMO-8 ->LOMO+1	16.38%
		HOMO-8 ->LOMO+2	11.53%
		HOMO-7 ->LOMO+1	25.47%
		HOMO-2 ->LOMO+3	15.35%
		HOMO-2 ->LOMO+4	21.42%
		HOMO-1 ->LOMO+3	22.26%
		HOMO-1 ->LOMO+4	25.05%
		HOMO-1 ->LOMO+5	26.47%
		HOMO-1 ->LOMO+8	10.57%
		HOMO ->LOMO+11	19.75%

Table S2. Calculated ($\lambda_{TD\text{-}DFT}$) wavelengths of **4b**. Molecular orbitals (MOs) involved in the main electronic transition，f corresponds to the oscillator strength. (TD-DFT, B3LYP-D3/6-31g(d))

$\lambda_{TD\text{-}DFT}$	Oscillator Strength, f	MOs	
381.69	0.3731	HOMO-1 ->LOMO	18.37%
		HOMO ->LOMO+1	67.26%
348.08	0.2702	HOMO-1 ->LOMO	65.17%
		HOMO ->LOMO+1	18.15%
		HOMO ->LOMO+2	13.38%
328.39	0.1042	HOMO-2 ->LOMO	29.32%
		HOMO ->LOMO+2	59.14%
		HOMO ->LOMO+3	15.72%
304.90	0.1052	HOMO-3 ->LOMO	59.70%
		HOMO ->LOMO+3	31.43%
284.39	0.6227	HOMO-6 ->LOMO	10.37%
		HOMO-5 ->LOMO	13.23%
		HOMO-1 ->LOMO+1	60.07%
		HOMO ->LOMO+4	22.75%
		HOMO ->LOMO+8	12.08%
267.64	0.1308	HOMO-7 ->LOMO	11.68%
		HOMO-2 ->LOMO+1	63.02%
		HOMO ->LOMO+3	10.68%
		HOMO ->LOMO+9	16.27%
261.55	0.1897	HOMO-10 ->LOMO	18.91%
		HOMO-1 ->LOMO+1	10.06%
		HOMO ->LOMO+8	19.42%
		HOMO ->LOMO+10	58.63%
261.23	0.1078	HOMO-10 ->LOMO	18.17%
		HOMO-2 ->LOMO+1	16.94%
		HOMO-1 ->LOMO+2	19.93%
		HOMO ->LOMO+8	15.20%
		HOMO ->LOMO+9	56.46%
251.33	0.1833	HOMO-10 ->LOMO	27.46%
		HOMO-9 ->LOMO	11.38%
		HOMO-8 ->LOMO	27.01%
		HOMO-3 ->LOMO+1	10.15%
		HOMO-2 ->LOMO+2	49.81%
		HOMO ->LOMO+10	13.64%

Table S3. Calculated ($\lambda_{TD\text{-}DFT}$) wavelengths of **4c**. Molecular orbitals (MOs) involved in the main electronic transition，f corresponds to the oscillator strength. (TD-DFT, B3LYP-D3/6-31g(d))

$\lambda_{TD\text{-}DFT}$	Oscillator Strength, f	MOs	
397.96	0.3393	HOMO-1 ->LOMO	13.10%
		HOMO ->LOMO+1	68.29%
341.40	0.0926	HOMO-1 ->LOMO	43.94%
		HOMO ->LOMO+1	10.83%
		HOMO ->LOMO+2	50.11%
		HOMO ->LOMO+3	12.61%
335.41	0.1719	HOMO-1 ->LOMO	51.03%
		HOMO ->LOMO+2	45.08%
297.77	0.1729	HOMO-5 ->LOMO	50.31%
		HOMO-4 ->LOMO	42.46%
		HOMO-2 ->LOMO	15.84%
289.25	0.4525	HOMO-1 ->LOMO+1	52.83%
		HOMO ->LOMO+4	25.73%
		HOMO ->LOMO+8	26.68%
		HOMO ->LOMO+9	14.36%
		HOMO ->LOMO+10	13.22%
285.24	0.2054	HOMO-1 ->LOMO+1	25.90%
		HOMO ->LOMO+4	25.57%
		HOMO ->LOMO+5	12.02%
		HOMO ->LOMO+7	16.28%
		HOMO ->LOMO+8	45.26%
		HOMO ->LOMO+9	22.35%
		HOMO ->LOMO+10	19.91%
257.39	0.1254	HOMO-8 ->LOMO	14.15%
		HOMO-3 ->LOMO+1	14.09%
		HOMO ->LOMO+8	28.47%
		HOMO ->LOMO+9	10.56%
		HOMO ->LOMO+10	55.01%
232.23	0.1095	HOMO-11 ->LOMO	22.59%
		HOMO-9 ->LOMO	11.65%
		HOMO-7 ->LOMO+1	14.92%
		HOMO-4 ->LOMO+2	55.30%
		HOMO-1 ->LOMO+3	15.74%

Table S4. Calculated (λ_{TD-DFT}) wavelengths of **4d**. Molecular orbitals (MOs) involved in the main electronic transition，f corresponds to the oscillator strength. (TD-DFT, B3LYP-D3/6-31g(d))

λ_{TD-DFT}	Oscillator Strength, f	MOs	
410.94	0.4496	HOMO ->LOMO	69.69%
292.71	0.3280	HOMO-1 ->LOMO	34.75%
		HOMO ->LOMO+5	24.70%
		HOMO ->LOMO+6	15.46%
		HOMO ->LOMO+7	28.07%
		HOMO ->LOMO+8	32.57%
		HOMO ->LOMO+9	26.59%
		HOMO ->LOMO+10	10.45%
287.73	0.7070	HOMO-1 ->LOMO	42.65%
		HOMO ->LOMO+5	23.40%
		HOMO ->LOMO+6	28.69%
		HOMO ->LOMO+8	32.99%
		HOMO ->LOMO+9	22.37%
287.68	0.1750	HOMO-1 ->LOMO	20.00%
		HOMO ->LOMO+7	62.54%
		HOMO ->LOMO+8	15.37%
		HOMO ->LOMO+9	13.54%
264.11	0.1440	HOMO-2 ->LOMO	34.22%
		HOMO ->LOMO+8	34.15%
		HOMO ->LOMO+9	46.32%
263.11	0.1457	HOMO-2 ->LOMO	50.95%
		HOMO ->LOMO+8	20.60%
		HOMO ->LOMO+9	19.30%
		HOMO ->LOMO+10	32.70%
258.84	0.1841	HOMO-6 ->LOMO	13.65%
		HOMO-2 ->LOMO	29.12%
		HOMO-1 ->LOMO+2	12.22%
		HOMO ->LOMO+9	18.19%
		HOMO ->LOMO+10	54.48%
234.14	0.2610	HOMO-3 ->LOMO	18.70%
		HOMO-2 ->LOMO+2	60.76%
		HOMO-1 ->LOMO+5	20.89%
		HOMO ->LOMO+11	10.63%

Table S5. Calculated ($\lambda_{TD\text{-}DFT}$) wavelengths of **4a-F**. Molecular orbitals (MOs) involved in the main electronic transition，f corresponds to the oscillator strength. (TD-DFT, B3LYP-D3/6-31g(d))

$\lambda_{TD\text{-}DFT}$	Oscillator Strength, f	MOs	
444.11	0.4133	HOMO ->LOMO	69.42%
378.56	0.1029	HOMO ->LOMO+1	69.19%
300.91	0.2392	HOMO-4 ->LOMO	35.51%
		HOMO-3 ->LOMO	44.09%
		HOMO-2 ->LOMO	24.15%
		HOMO-1 ->LOMO	15.22%
		HOMO ->LOMO+8	24.78%
282.27	0.1763	HOMO-4 ->LOMO	31.73%
		HOMO ->LOMO+7	21.57%
		HOMO ->LOMO+8	56.38%
267.13	0.1722	HOMO-6 ->LOMO	65.74%
		HOMO ->LOMO+10	11.58%
		HOMO ->LOMO+11	12.48%
253.28	0.3580	HOMO-6 ->LOMO+1	63.82%
		HOMO-4 ->LOMO+2	13.77%
		HOMO-3 ->LOMO+3	11.48%
		HOMO-1 ->LOMO+4	14.38%
252.64	0.0174	HOMO-6 ->LOMO+1	12.09%
		HOMO-4 ->LOMO+3	43.97%
		HOMO-3 ->LOMO+2	10.28%
		HOMO-3 ->LOMO+3	51.31%
251.30	0.0158	HOMO-8 ->LOMO	14.26%
		HOMO-6 ->LOMO+1	10.68%
		HOMO-6 ->LOMO+2	16.88%
		HOMO-1 ->LOMO+4	59.63%
249.96	0.0194	HOMO-11 ->LOMO	16.25%
		HOMO-11 ->LOMO+1	13.20%
		HOMO-9 ->LOMO+2	12.13%
		HOMO-6 ->LOMO+1	12.78%
		HOMO-6 ->LOMO+2	49.26%
		HOMO-6 ->LOMO+3	12.38%
		HOMO-5 ->LOMO+2	13.92%
		HOMO-4 ->LOMO+2	28.58%
		HOMO-1 ->LOMO+4	16.47%

Table S6. Calculated ($\lambda_{TD\text{-}DFT}$) wavelengths of **4b-F**. Molecular orbitals (MOs) involved in the main electronic transition，f corresponds to the oscillator strength. (TD-DFT, B3LYP-D3/6-31g(d))

$\lambda_{TD\text{-}DFT}$	Oscillator Strength, f	MOs	
430.55	0.5816	HOMO ->LOMO	69.91%
309.09	0.1320	HOMO-2 ->LOMO	10.16%
		HOMO-1 ->LOMO	50.63%
		HOMO ->LOMO+5	22.89%
		HOMO ->LOMO+6	35.31%
		HOMO ->LOMO+7	15.53%
293.97	0.2665	HOMO-2 ->LOMO	29.01%
		HOMO-1 ->LOMO	14.43%
		HOMO ->LOMO+4	55.54%
		HOMO ->LOMO+5	17.86%
		HOMO ->LOMO+6	17.02%
283.29	0.1595	HOMO-5 ->LOMO	10.84%
		HOMO-3 ->LOMO	18.44%
		HOMO-2 ->LOMO	14.69%
		HOMO ->LOMO+5	51.01%
		HOMO ->LOMO+6	34.76%
		HOMO ->LOMO+7	16.30%
275.58	0.1641	HOMO-4 ->LOMO	28.89%
		HOMO-1 ->LOMO+1	23.62%
		HOMO ->LOMO+6	19.25%
		HOMO ->LOMO+7	51.10%
268.82	0.1561	HOMO-7 ->LOMO	20.66%
		HOMO-5 ->LOMO	60.65%
		HOMO-1 ->LOMO+2	19.90%
256.46	0.1445	HOMO-7 ->LOMO	21.29%
		HOMO-3 ->LOMO+1	38.80%
245.21	0.2197	HOMO-5 ->LOMO+1	56.60%
		HOMO-5 ->LOMO+2	15.18%
		HOMO-3 ->LOMO+2	24.33%
		HOMO-3 ->LOMO+3	12.48%
		HOMO-2 ->LOMO+2	10.76%
		HOMO-2 ->LOMO+3	16.35%

Table S7. Calculated ($\lambda_{TD\text{-}DFT}$) wavelengths of **4c-F**. Molecular orbitals (MOs) involved in the main electronic transition，f corresponds to the oscillator strength. (TD-DFT, B3LYP-D3/6-31g(d))

$\lambda_{TD\text{-}DFT}$	Oscillator Strength, f	MOs	
467.46	0.5582	HOMO ->LOMO	70.06%
317.61	0.2685	HOMO-3 ->LOMO	67.31%
		HOMO ->LOMO+3	10.65%
309.61	0.0905	HOMO-5 ->LOMO	10.54%
		HOMO-4 ->LOMO	67.41%
289.77	0.1200	HOMO-6 ->LOMO	11.81%
		HOMO-1 ->LOMO+1	27.11%
		HOMO ->LOMO+4	53.75%
		HOMO ->LOMO+5	11.92%
		HOMO ->LOMO+6	26.02%
287.82	0.0856	HOMO-7 ->LOMO	14.20%
		HOMO-6 ->LOMO	64.22%
		HOMO-3 ->LOMO+1	20.64%
		HOMO ->LOMO+4	10.28%
279.26	0.1038	HOMO-4 ->LOMO+1	13.95%
		HOMO-3 ->LOMO+1	33.61%
		HOMO ->LOMO+5	46.00%
		HOMO ->LOMO+6	33.09%
262.15	0.1038	HOMO-7 ->LOMO	53.47%
		HOMO-6 ->LOMO	12.05%
		HOMO-6 ->LOMO+1	33.30%
		HOMO-3 ->LOMO+3	13.24%
		HOMO-1 ->LOMO+2	11.88%
260.48	0.1574	HOMO-7 ->LOMO	28.63%
		HOMO-6 ->LOMO+1	56.62%
		HOMO-1 ->LOMO+2	13.26%
		HOMO ->LOMO+8	18.70%

4. Aggregation-induced fluorescence properties

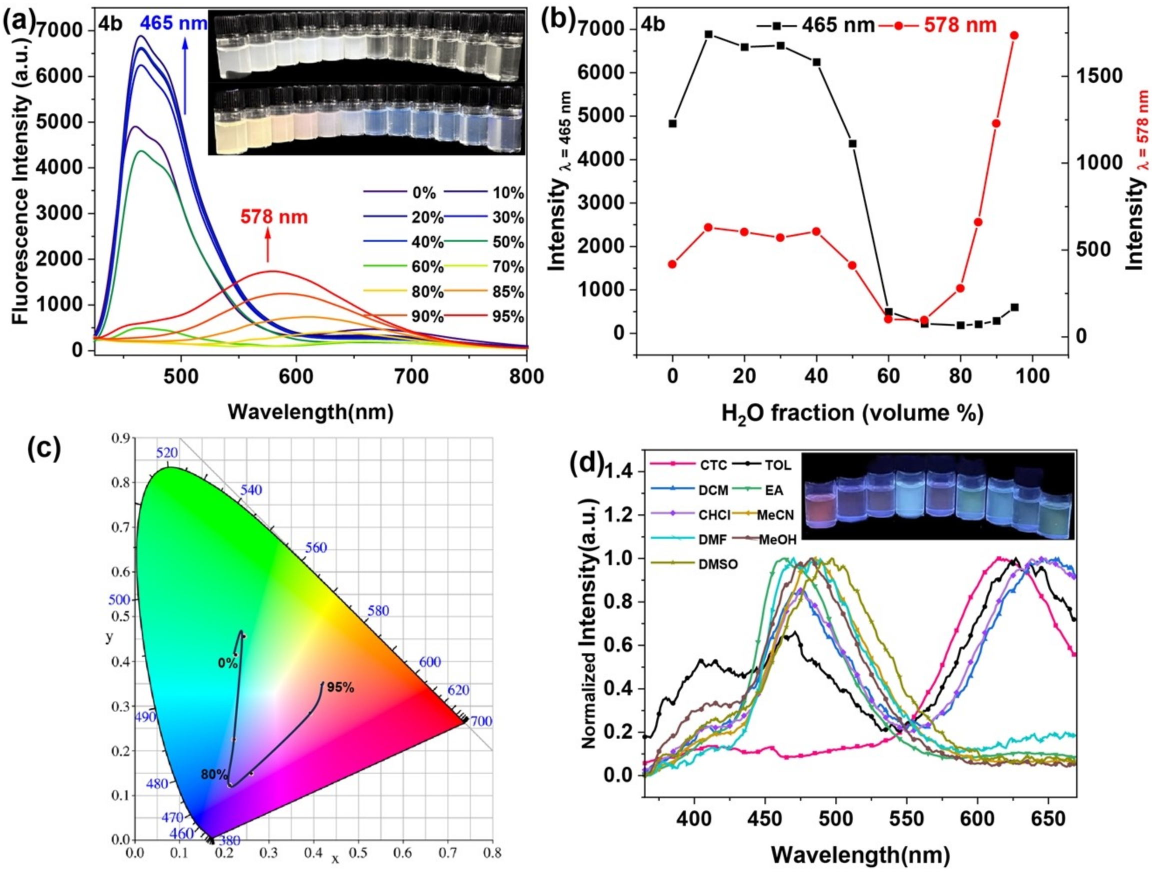

Figure S10. Photoluminescence (PL) spectra of **4b** in different THF/water ratios. (a) Photographs of aggregates under UV light, λ_{ex} = 365 nm. (b) Emission intensity of **4b** as the THF/water ratio is altered (Left: fluorescence intensity at 465 nm; Right: fluorescence intensity at 578 nm). (c) CIE chromaticity. (d) Emission spectra of **4b** in solutions (with different polarities).

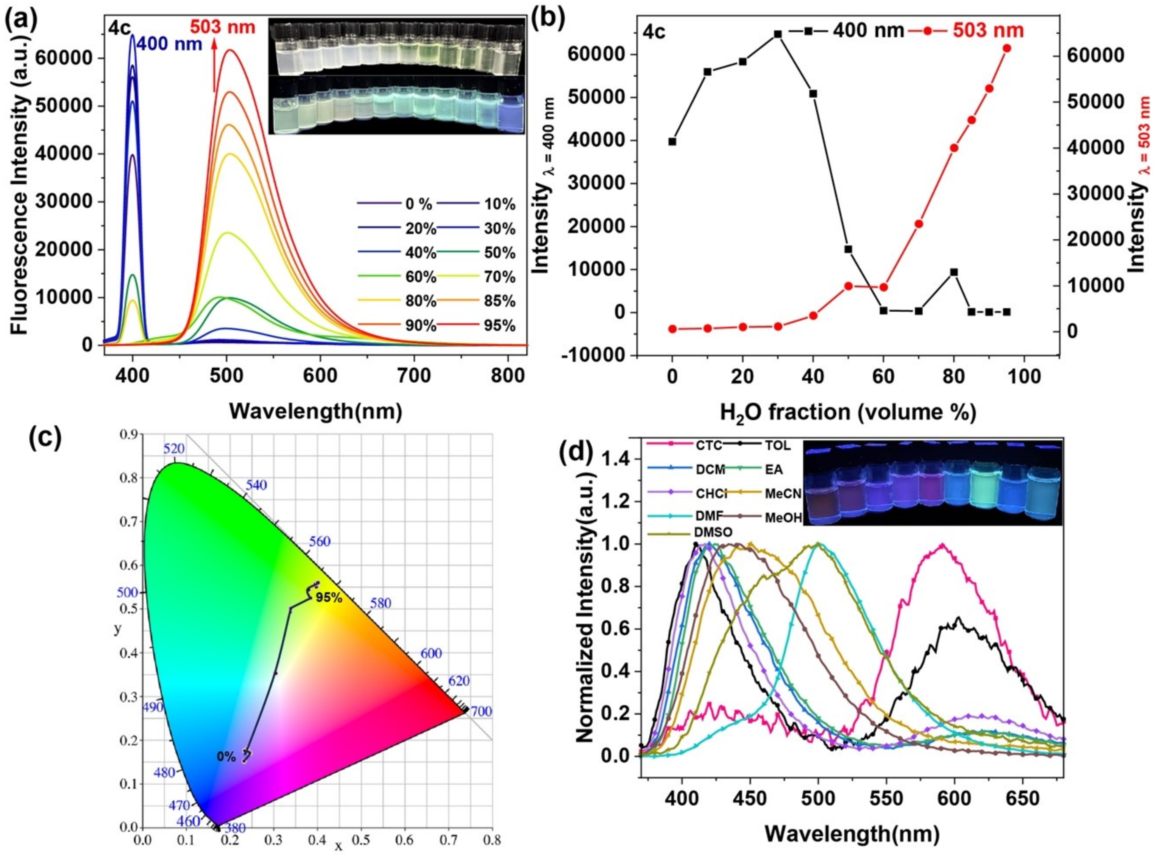

Figure S11. Photoluminescence (PL) spectra of **4c** in different THF/water ratios. (a) Photographs of aggregates under UV light, λ_{ex} = 365 nm. (b) Emission intensity of **4c** as the THF/water ratio is altered (Left: fluorescence intensity at 400 nm; Right: fluorescence intensity at 503 nm). (c) CIE chromaticity. (d) Emission spectra of **4c** in solutions (with different polarities).

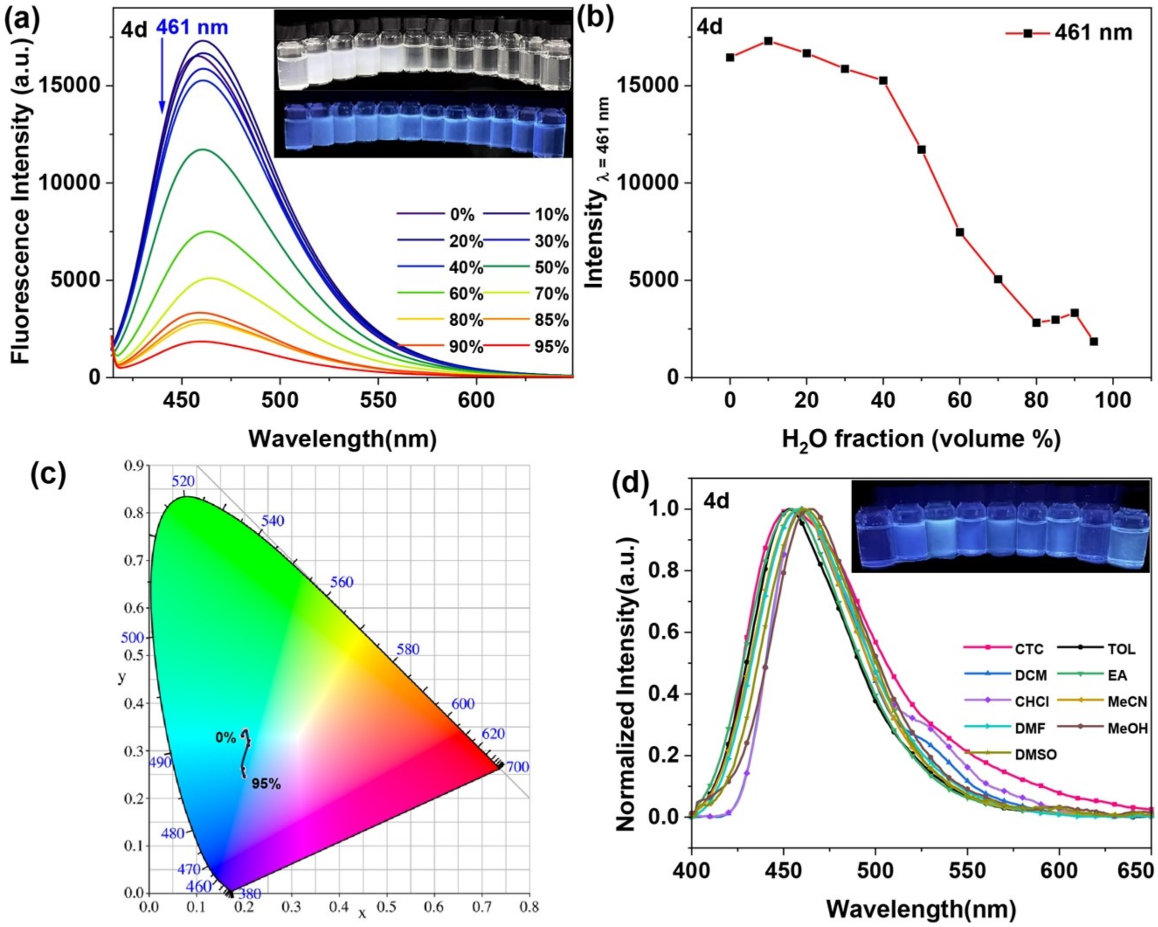

Figure S12. Photoluminescence (PL) spectra of **4d** in different THF/water ratios. (a) Photographs of aggregates under UV light, λ_{ex} = 365 nm. (b) Emission intensity of **4d** as the THF/water ratio is altered (Left: fluorescence intensity at 465 nm; Right: fluorescence intensity at 578 nm). (c) CIE chromaticity. (d) Emission spectra of **4d** in solutions (with different polarities).

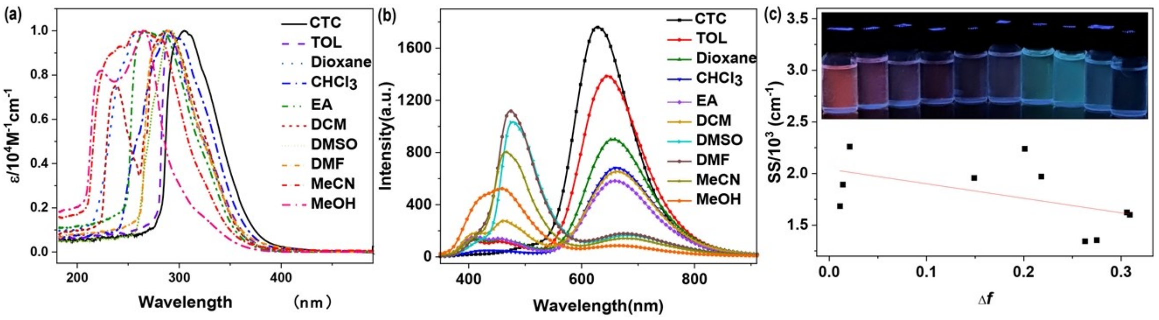

Figure S13. Figure Sx. (a) absorption and (b) emission spectra of 4a in different solvents; (c) Plots of Stokes shift (SS) of 4a versus solvent polarity parameter (Δf).

Table S8. Photophysical Properties of **4a** in Different Solvents

Solvent	Δf [a]	λ_{ab} (nm) [b]	λ_{em} (nm) [c]	SS [d]
CCl_4	0.011	305	627	1684
Toluene	0.014	290	643	1893
dioxane	0.021	264	655	2261
chloroform	0.149	288	660	1957
EA	0.201	266	658	2240
DCM	0.218	287	661	1971
DMSO	0.263	291	478	1344
DMF	0.275	289	475	1355
acetonitrile	0.306	265	465	1623
Methanol	0.309	265	460	1600

[a] solvent polarity parameter taken from the Lippert-Mataga equation. [b] The longest λ_{max} values in the UV-Vis spectra. [c] Emission maxima in solvent. [d] Stokes shift = $1/\lambda_{abs} - 1/\lambda_{em}$.

5. Electrochemical properties

Cyclic voltammograms were recorded with a Metrohm PGSTAT204 electrochemical analyzer using DCM. The CV cell consisted of a gold electrode, a Pt wire counter electrode, and an Ag/AgCl reference electrode. All measurements were performed using DCM solutions of samples with a concentration of 1 mM and 0.1 M $Bu_4N^+BF_6^-$ as a supporting electrolyte with a scan rate of 100 mVs^{-1}. Potentials are determined against a ferrocene/ferrocenyl ion couple (Fc/Fc^+).

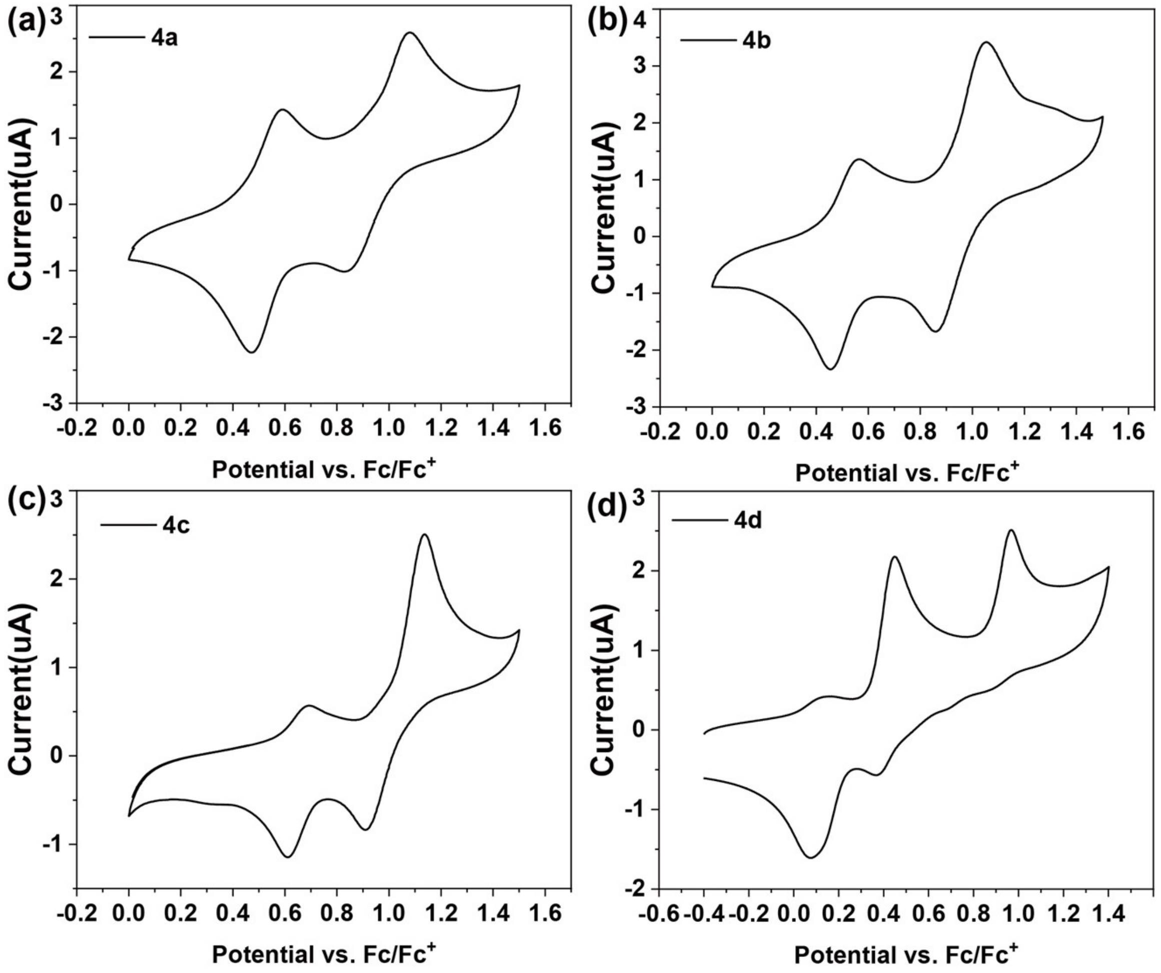

Figure S14. Cyclic voltammograms of **4a**, **4b**,**4c** and **4d** in DCM with $Bu_4N^+BF_6^-$ (0.1 M) as a supporting electrolyte, Fc = ferrocene.

6. Comparison of HOMO/LUMO plots

Table S9. Electronic properties of **4a**, **4b**, **4c** and **4d**

Entry	E_g[a](eV)	E_{OX}[b](V)	HOMO(eV) (Exp)[c]	LUMO(eV) (Exp)[d]	HOMO(eV) (Cal)[e]	LUMO(eV) (Cal)[e]	E_g (eV) (Cal)[e]
**4a**	3.59	0.79	-5.59	-2.00	-5.40	-0.95	4.46
**4b**	3.55	0.86	-5.66	-2.11	-5.30	-0.85	4.45
**4c**	3.40	0.96	-5.76	-2.36	-5.74	-1.45	4.29
**4d**	3.20	0.78	-5.58	-2.38	-4.88	-0.79	4.09

[a] E_g estimated from the UV-Vis absorption spectra.

[b] Oxidation onset potentials measured by cyclic voltammetry.

[c] HOMO = $-(E_{OX} + 4.8)$ eV.

[d] LUMO = HOMO + E_g.

[e]Theoretical calculations have been carried out by using the GAUSSIAN09 suite of programs in gas-phase at the B3LYP/6-31G(d) levele,[5] respectively.

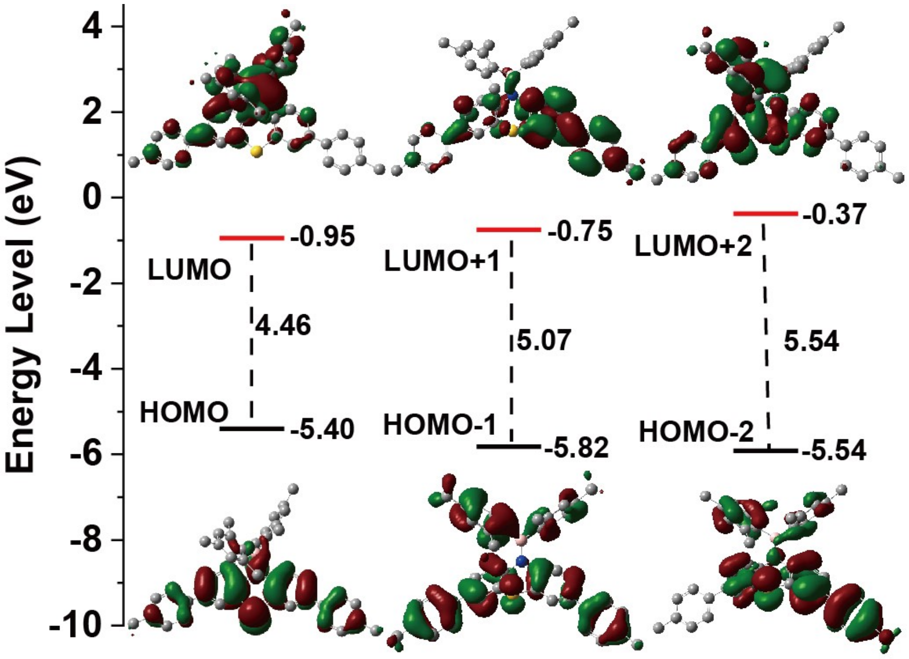

Figure S15. Computed molecular orbital plots for **4a**.

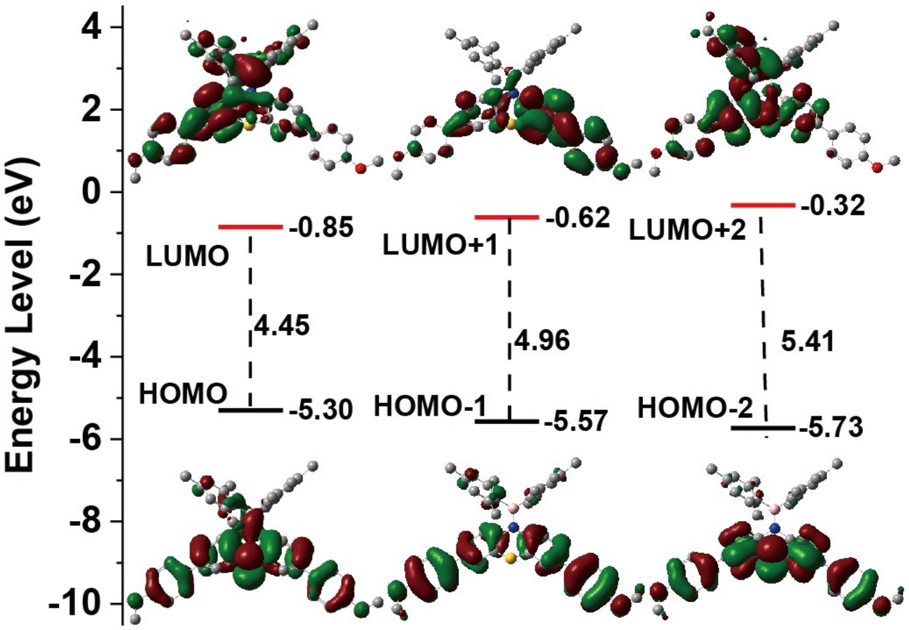

Figure S16. Computed molecular orbital plots for **4b**.

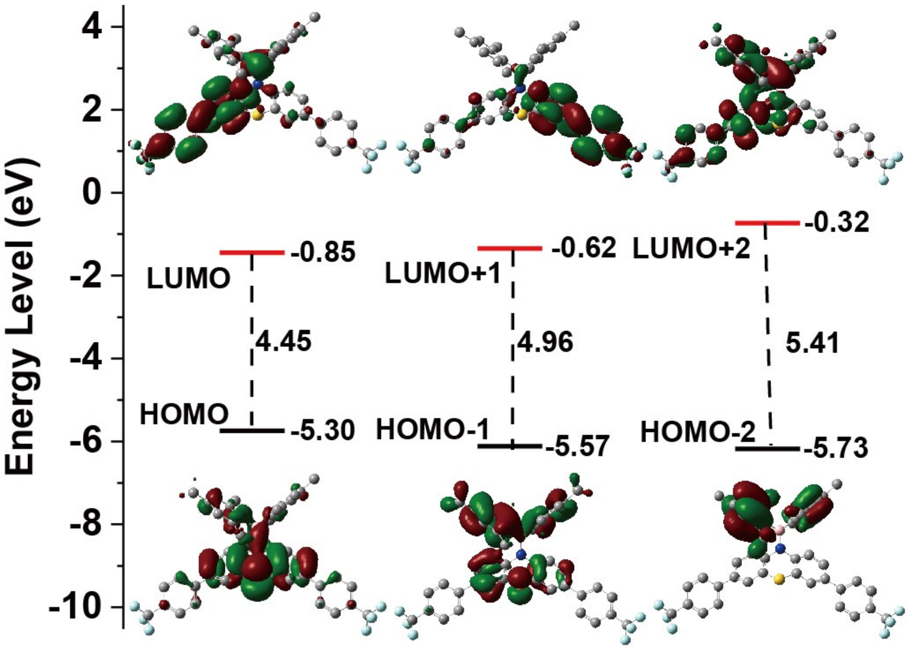

Figure S17. Computed molecular orbital plots for **4c**.

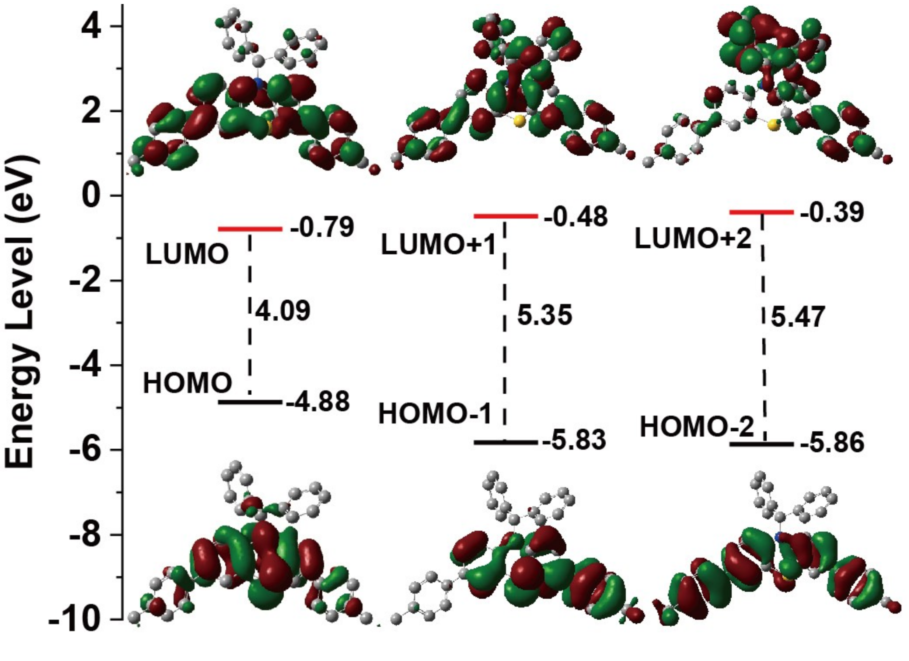

Figure S18. Computed molecular orbital plots for **4d**.

7. Determination of the detection limit

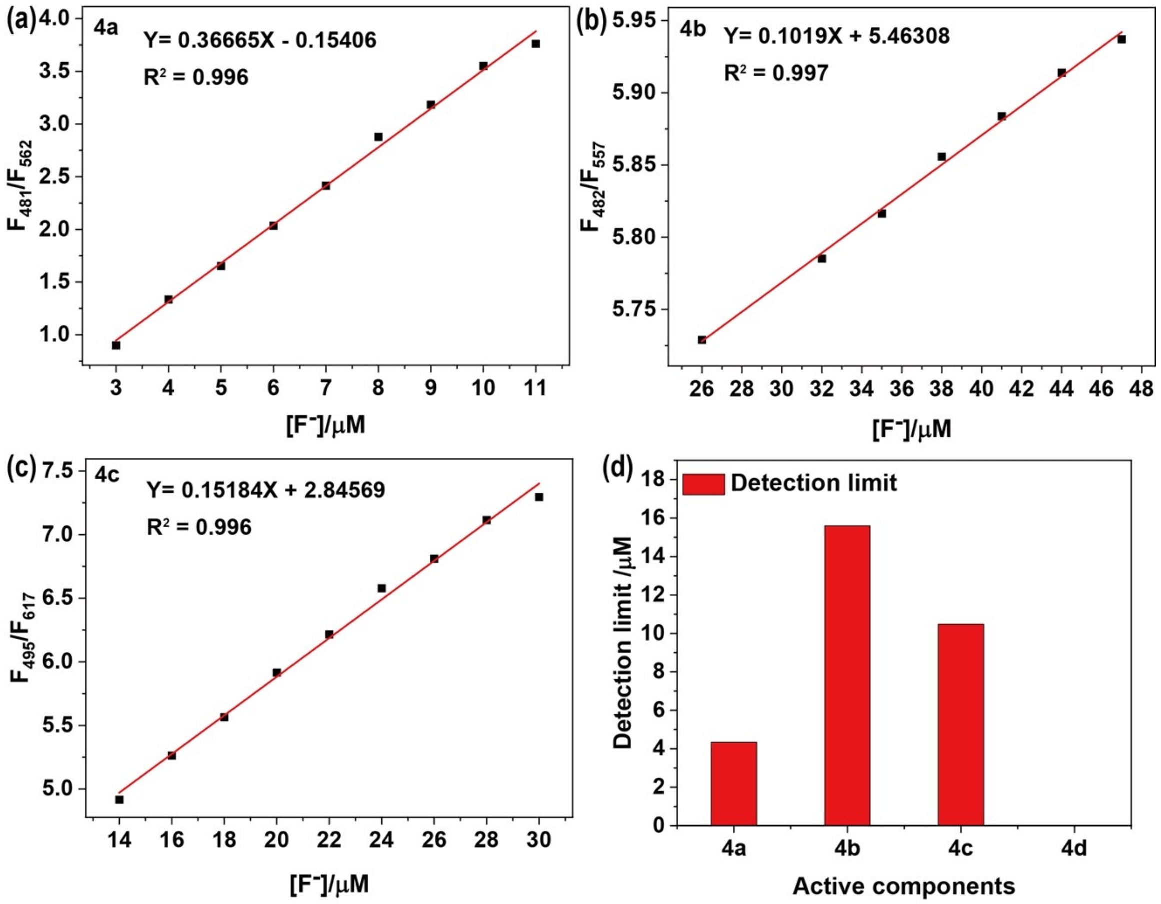

Figure S19. (a-c) The calibration curves and linear equation of **4a-4c** for FL intensity ratio changes upon gradual addition of F^-. (d)The calculated detection limits of **4a**, **4b**, and **4c**.

8. Reversibility of the MFC performance

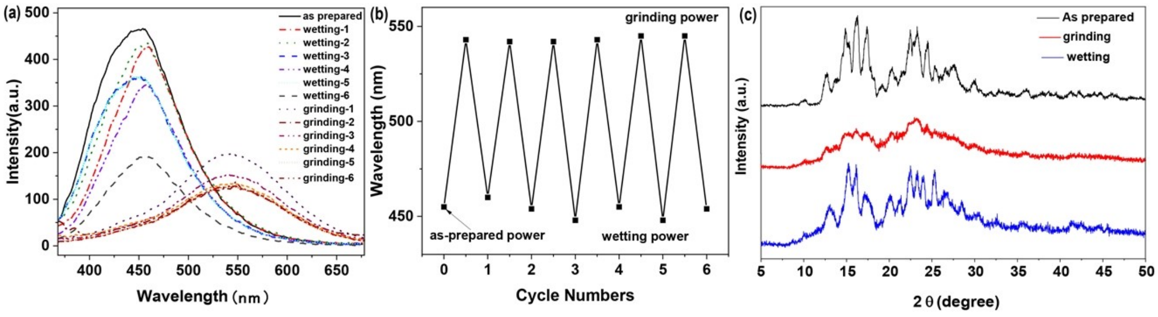

Figure S20. Emission spectra (a) and maximum emission wavelengths of **4a** upon repeating treated by grinding and wetting with DCM. (c) XRD patterns of **4a**: as-prepared, grinding and wetted samples.

9. ^{1}H NMR and ^{13}C NMR spectra

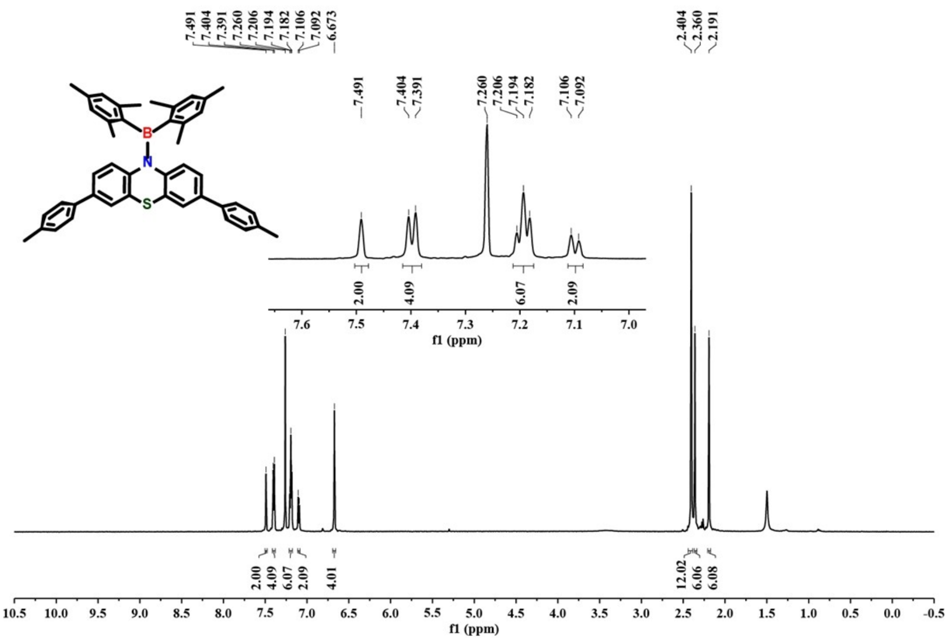

^{1}H NMR spectrum of **4a** in $CDCl_3$

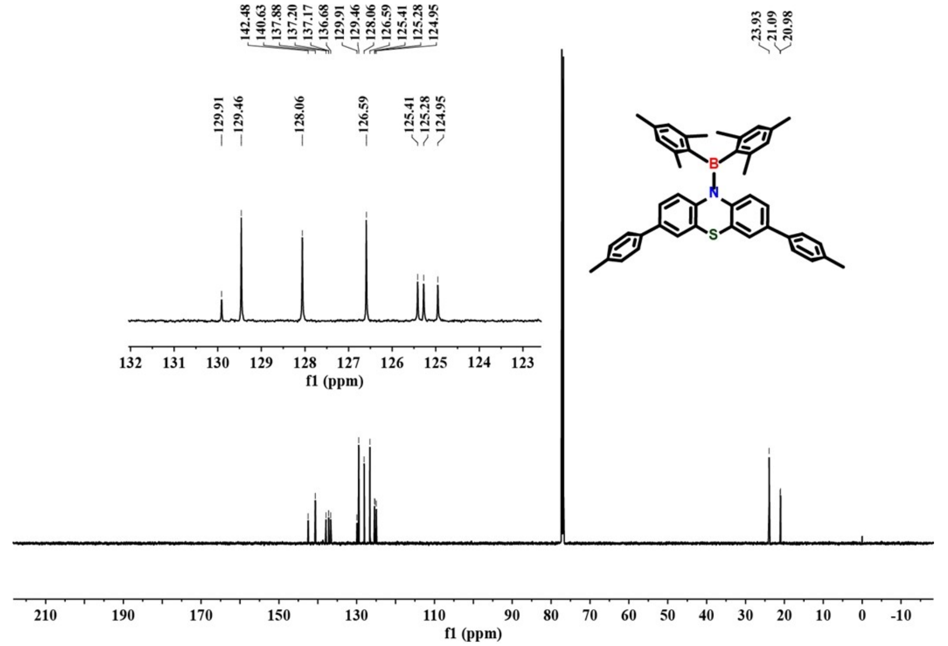

^{13}C NMR spectrum of **4a** in $CDCl_3$

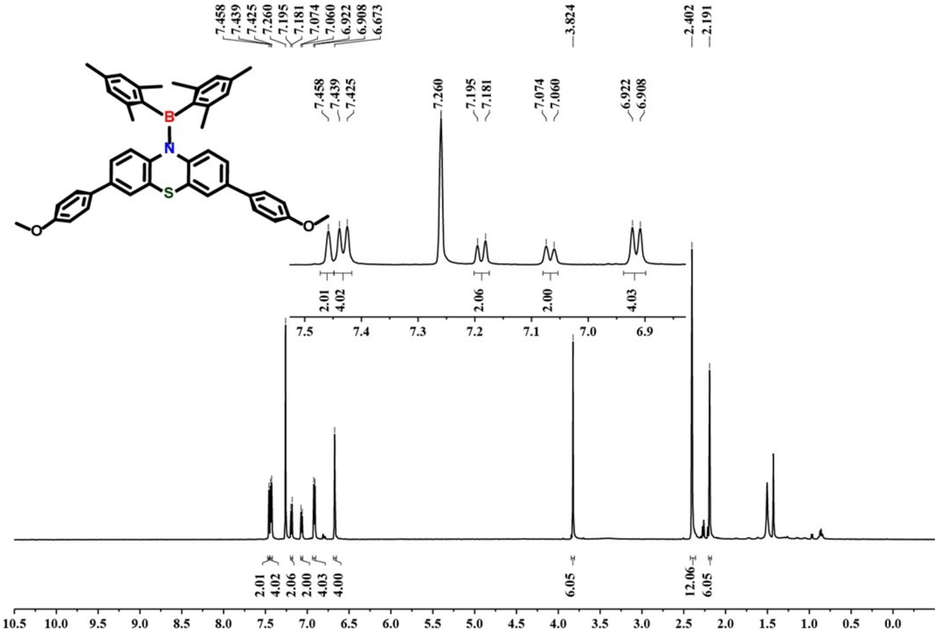

^{1}H NMR spectrum of **4b** in $CDCl_3$

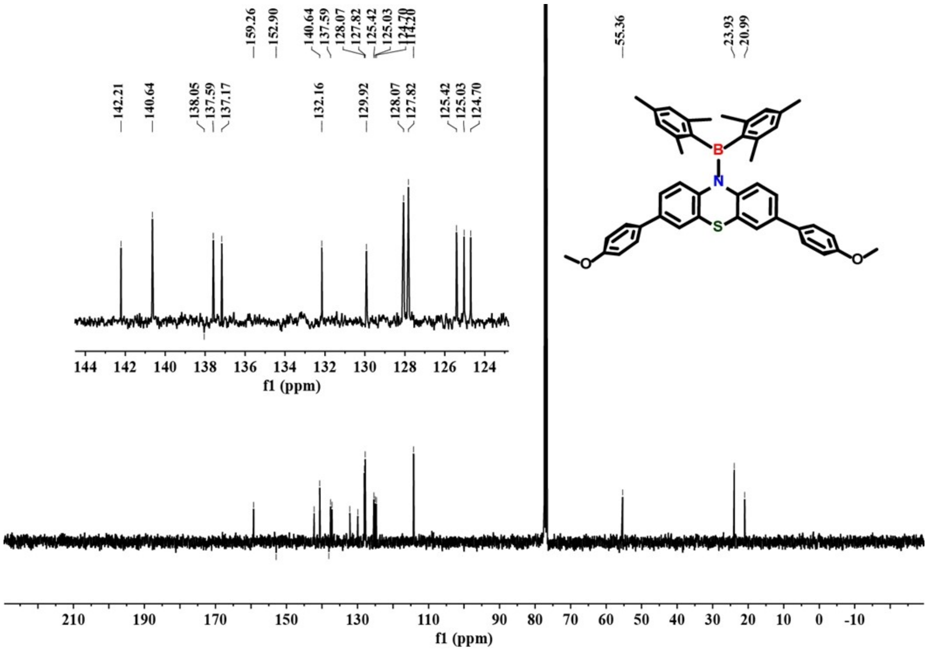

^{13}C NMR spectrum of **4b** in $CDCl_3$

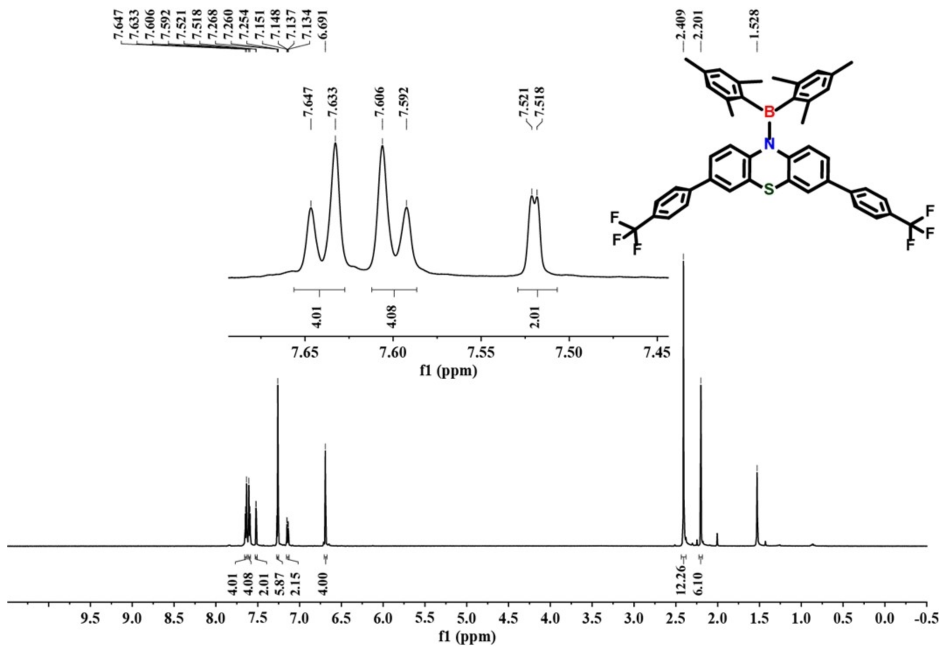

^{1}H NMR spectrum of **4c** in $CDCl_3$

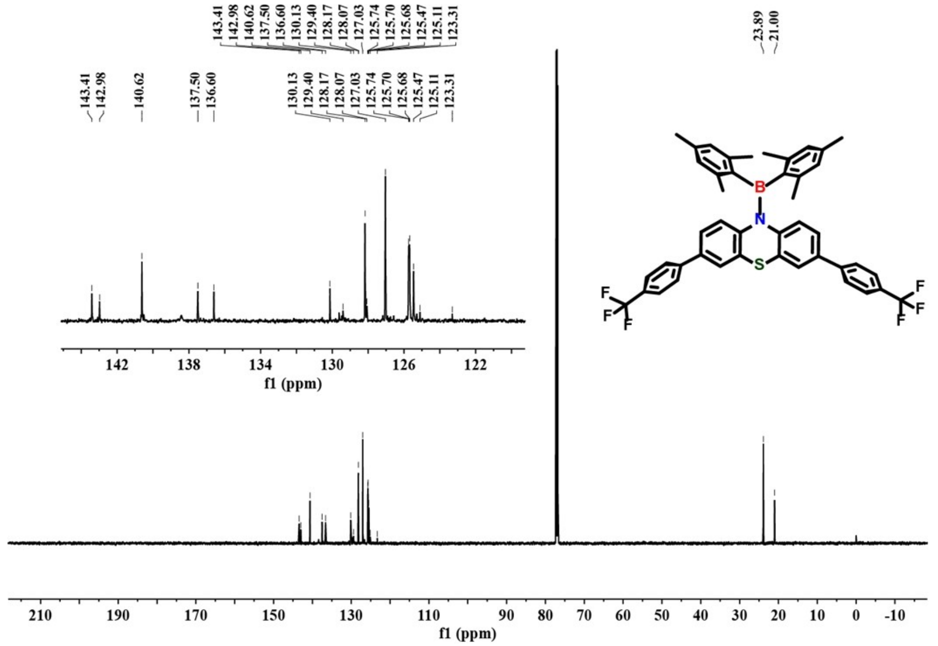

^{13}C NMR spectrum of **4c** in $CDCl_3$

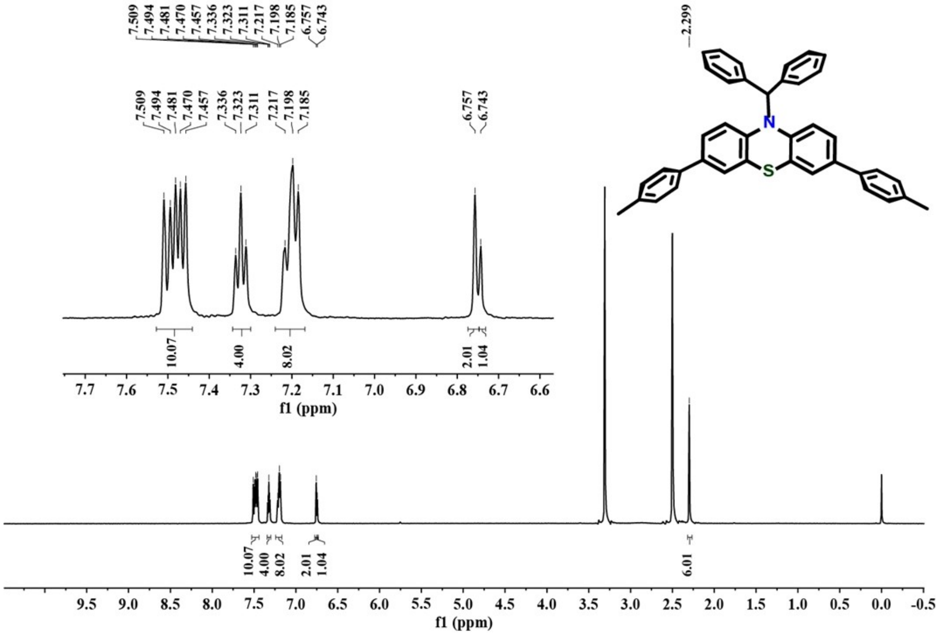

^{1}H NMR spectrum of **4d** in DMSO

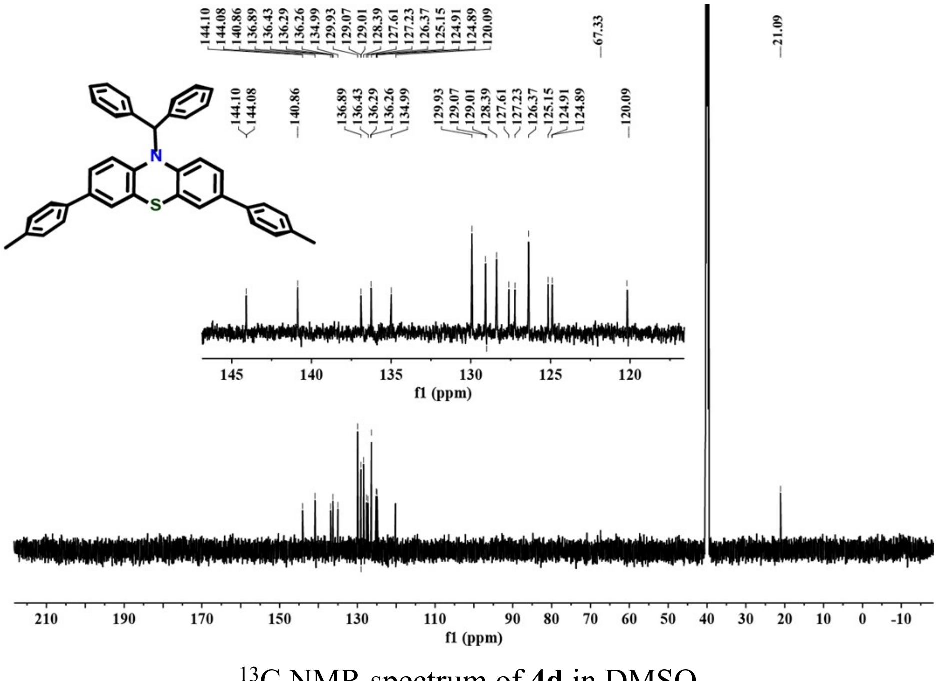

^{13}C NMR spectrum of **4d** in DMSO

10. HRMS spectra

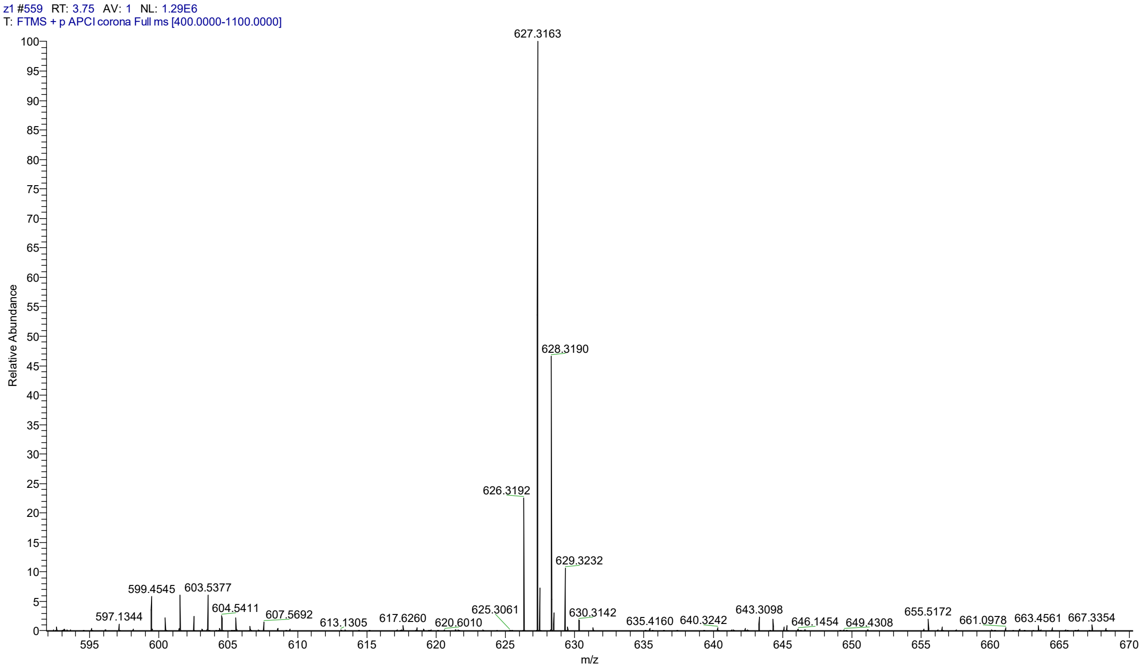

HRMS of **4a**

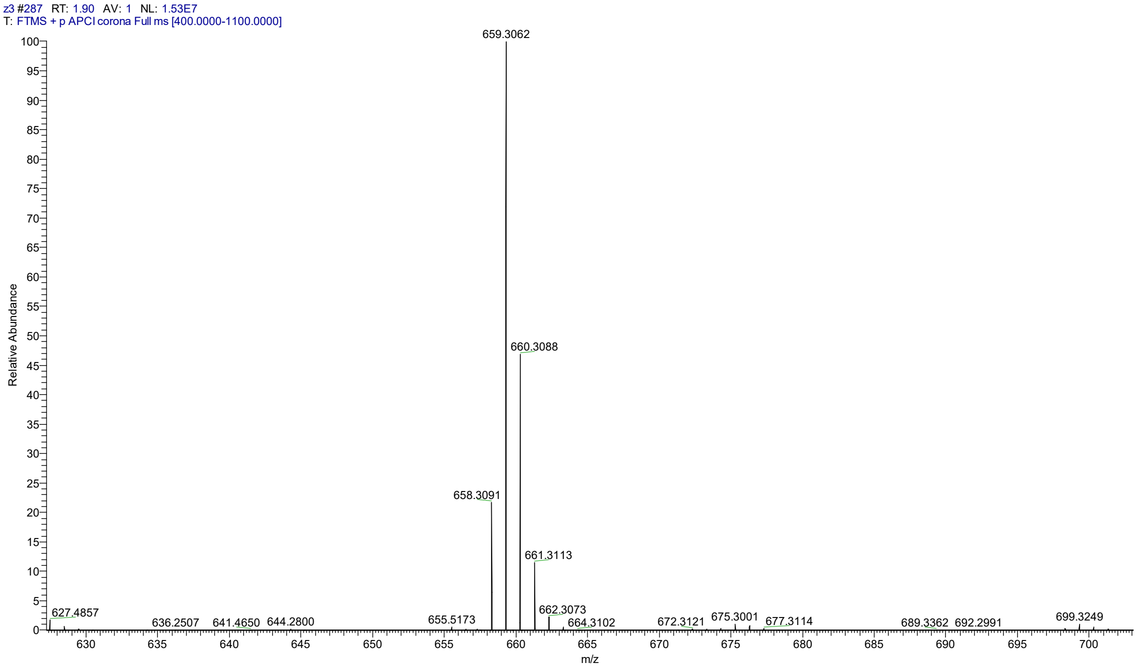

HRMS of **4b**

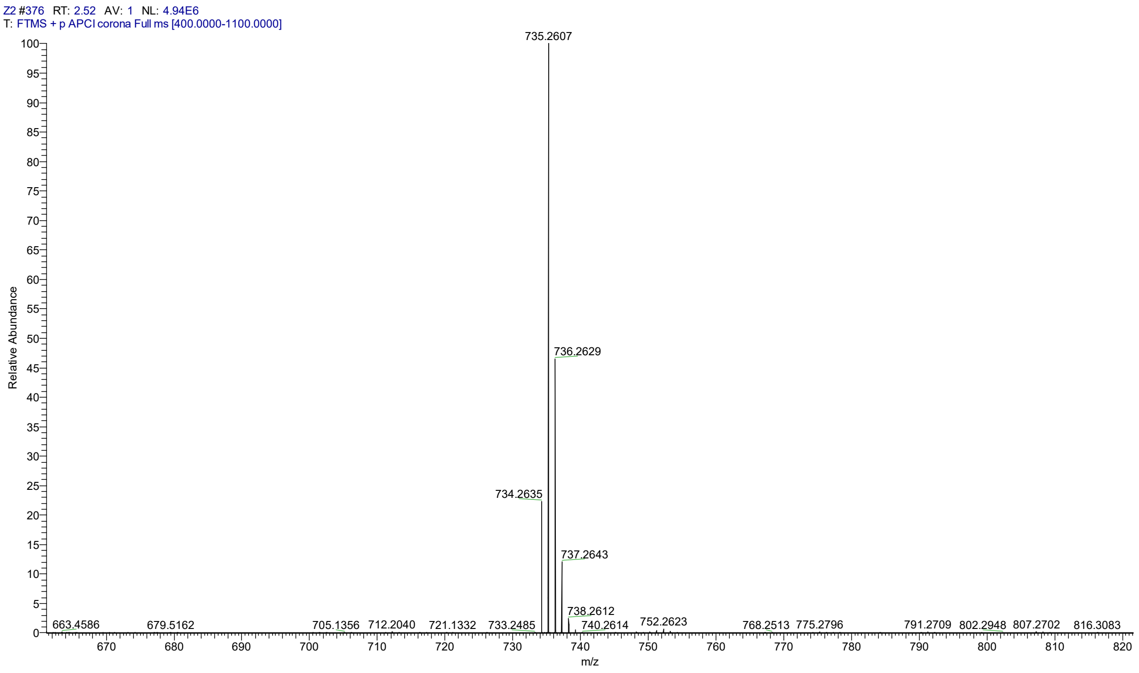

HRMS of **4c**

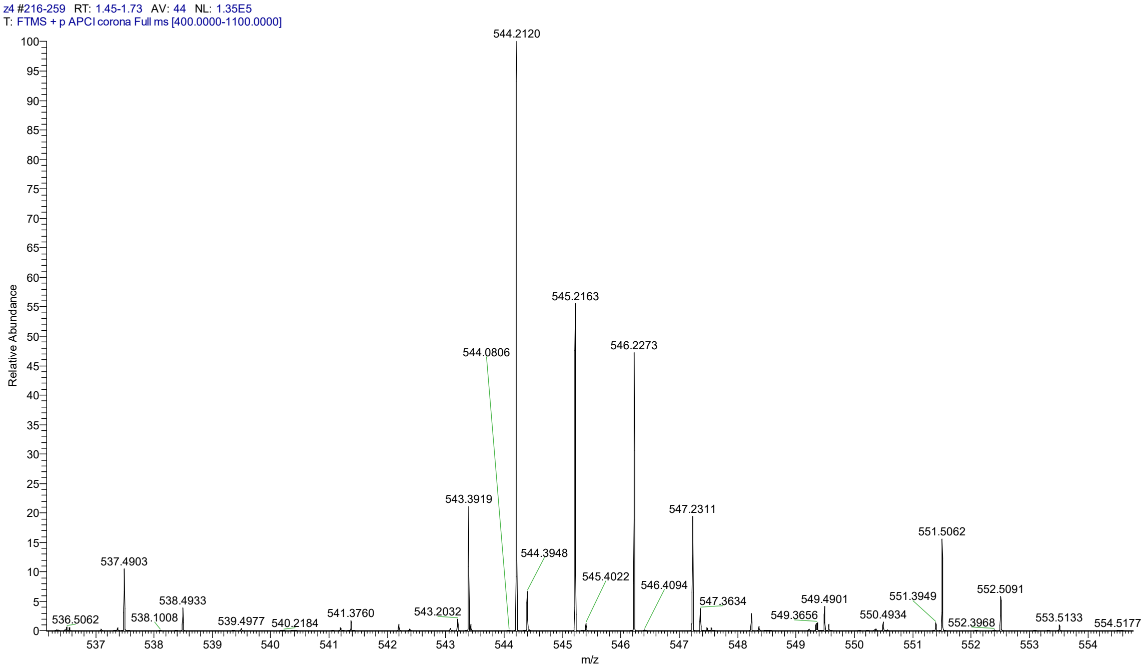

HRMS of **4d**

11. Reference

(1) Miertusˇ, S.; Scrocco, E.; Tomasi, J. *Chem. Phys.* **1981**, *55*, 117.

(2) Cammi, R.; Tomasi, J. J. *Comput. Chem.* **1995**, *16*, 1449.

(3) Tomasi, J.; Persico, M. *Chem. Rev*. **1994**, *94*, 2027.

(4) Cramer,C. J.; Truhlar, D. G. *Chem. Rev*. **1999**, *99*, 2161.

(5) Frisch, M. J.; Trucks, G. W.; Schlegel, H. B. Gaussian09, Revision A.01, Gaussian, Wallingford, Con, USA, **2009**.

Author Contributions

Weidong Zhang conceived the idea for the study. Guoqiang Li prepared the samples and conducted characterizations. Guoqiang Li, Yan Wang, Zengheng Wen, and Zhuang Luo helped to prepare and characterize the samples. Weidong Zhang and Guoqiang Li wrote the manuscript and Guoqiang Li, Yan Wang, Yaohui Li, and Weijun Song revised and polished the manuscript.
